# Urinary Exosomal microRNA-451-5p Is a Potential Early Biomarker of Diabetic Nephropathy in Rats

**DOI:** 10.1371/journal.pone.0154055

**Published:** 2016-04-21

**Authors:** Aradhana Mohan, Ravi Shankar Singh, Manju Kumari, Devika Garg, Aditya Upadhyay, Carolyn M. Ecelbarger, Sucheta Tripathy, Swasti Tiwari

**Affiliations:** 1 Department of Molecular Medicine & Biotechnology, Sanjay Gandhi Postgraduate Institute of Medical Sciences, Lucknow, Uttar Pradesh, India; 2 Department of Medicine, Georgetown University, Washington, DC, United States of America; 3 Structural Biology and Bioinformatics Division, CSIR - Indian Institute of Chemical Biology, Kolkata, India; Emory University, UNITED STATES

## Abstract

Non-invasive renal signatures can help in serial monitoring of diabetic patients. We tested whether urinary exosomal (UE) microRNA (miR) analysis could non-invasively predict renal pathology in diabetic rats during the course of diabetes. Diabetes mellitus (DM) was induced in male Wistar rats by a single intraperitoneal injection of streptozotocin (STZ, 50 mg/kg body weight). Non-diabetic control (CTRL) rats were injected with vehicle. Insulin (INS) treatment (5U/d, s.c.) was provided to 50% of the DM rats. Urine samples were collected at weeks 3, 6, and 9 following injections and UE prepared. An increase in miR-451-5p and miR-16, observed by pilot small RNA sequencing of UE RNA, was confirmed by quantitative real-time polymerase chain reaction (qPCR) and selected for further study. Subsets of rats were euthanized after 3, 6, and 9 weeks of diabetes for renal pathology analysis, including determination of the tubulointerstitial fibrotic index (TFI) and glomerulosclerotic index (GI) scores. qPCR showed a substantial rise in miR-451-5p in UE from DM rats during the course of diabetes, with a significant rise (median fold change >1000) between 3 and 6 weeks. Moreover, UE miR-451-5p at 6 weeks predicted urine albumin at 9 weeks (r = 0.76). A delayed but significant rise was also observed for miR-16. In contrast, mean urine albumin only increased 21% between 3 and 6 weeks (non-significant rise), and renal TFI and GI were unchanged till 9 weeks. Renal expression of miR-451-5p and miR-16 (at 10 weeks) did not correlate with urine levels, and moreover, was negatively associated with indices of renal pathology (r≥-0.70, p = 0.005 for TFI and r≥-0.6, p≤0.02 for GI). Overall, a relative elevation in renal miR-451-5p and miR-16 in diabetes appeared protective against diabetes-induced kidney fibrosis; while UE miR-451-5p may hold prognostic value as an early and sensitive non-invasive indicator of renal disease.

## Introduction

Over one third of diabetic patients develop serious complications including nephropathy [1, 2]. A test for the rise in urine albumin levels (albuminuria) is routinely used for non-invasive serial monitoring of renal injury in these patients [3, 4]. However, it has limited ability to predict the earliest stages of diabetic nephropathy [5]. Early signatures such as microRNAs (miRNAs) have the potential to identify patients at risk and may improve disease prognosis. MiRNAs control mRNA expression of multiple genes and are thus, critical for many physiological processes including cell proliferation, cell differentiation, and cell death [6, 7]. MicroRNA are small (21–25 nucleotides) non-coding, single-stranded RNA molecules which are highly conserved [8]. They are endogenously produced and play significant role in the regulation of genes at a post-transcriptional level. They bind to the 3′-untranslated region (UTR) of the target mRNA, inducing its degradation and thereby, resulting in translational repression [9]. Due to their capability to regulate gene expression at the mRNA level, they serve as important upstream players in various cellular and physiological activities, including cell development, differentiation, proliferation, and apoptosis, and also in a variety of human diseases [10]. Moreover, miRNA mis-expression has been implicated in the pathogenesis of both diabetic, as well as, non-diabetic kidney diseases [11–18]. However, studies to examine the value of miRNA signatures for early diagnosis of diabetic nephropathy and to categorize subsets of diabetic patients that go on to develop overt nephropathy, a major clinical challenge, are lacking [11]. Moreover, kidney biopsy would not be considered the method of choice to serially monitor altered microRNA signatures in patients. In this light, exosomes in urine could prove helpful, as they have been shown to encapsulate biomolecules of renal origin including miRNAs [19, 20]. Exosomes are 30–100-nm intraluminal vesicles of multivesicular bodies (MVB). These are released upon exocytic fusion of the MVB with the plasma membrane and are increasingly recognized as a novel mode of cell-independent communication [21]. These tiny vesicles were recently discovered in urine by [20] and named, “urinary exosomes” (UE). Other than the presence of proteins, these vesicles are also enriched in mRNAs, microRNAs, and other non-coding RNAs [22]. Isolation of UE from total urine aids in enrichment of less-abundant biomolecules, including miRNAs, with a potentially high diagnostic value relative to the physiological and pathological state of the renal system [19, 23, 24]. Data on human UE reported by us and others, have suggested the usefulness of these vesicles as early non-invasive markers for diabetic nephropathy [23, 25].

Overall, microRNA analysis in urinary exosomes could lead to the discovery of new non-invasive biomarkers for early kidney disease [26, 27], and provide us with a better understanding of the biology underlying renal disease. For this, however, it is important to study the changes in urinary exosomal microRNA levels early on during the course of diabetes, before renal damage occurs, which forms the focus of our study. We conducted a detailed analysis of the time-course regulation of miR-451-5p and miR-16 in urinary exosomes and found them to be increased substantially on an initial screen of all urine exosomal miRNAs. We compared urinary exosomal expression of these two miRNAs to renal pathology, including albumin excretion, during the time course of type 1 diabetes in rats. We also tested how renal levels of these miRNAs are related to renal pathology.

## Materials and Methods

### Animal studies

All animal care and experimental procedures were approved by the Institutional Animal Ethics Committee (IAEC) of Sanjay Gandhi Postgraduate Institute of Medical Sciences (Registration no. 57/PO/ReBi/SL/99/CPCEA), according to guidelines from the Committee for the Purpose of Control and Supervision on Experiments on Animals (CPCSEA), Ministry of Environment and Forests, New Delhi, India. Male Wistar rats (*R*. *norvegicus*) (weight range 250–300 g, mean weight 261.3 ± 13.8 g, and, age range 90–105 days, mean age 96± 2days) were obtained from the Indian Institute of Toxicology Research, Lucknow, India. Animals were acclimatized under laboratory conditions for 2 weeks prior to experimentation. They were housed in standard rat cages with 3 companions in each cage in a temperature- (22–24°C) and humidity- (50–60%) controlled room with a 12-hour light/dark cycle. Animals had free access to a standard chow diet and water. For **study 1**, type I diabetes mellitus was induced in 16 rats via intraperitoneal administration of streptozotocin (STZ) (50mg/kg body weight of rat) dissolved in 0.1M citric acid buffer with pH 4.5 after 18 hrs of fasting (Sigma Chemical Co., St. Louis, MO, USA, as described previously [28, 29]. The non-diabetic control rats were given an intraperitoneal injection of vehicle (CTRL, n = 6). Following the STZ injection, rats were given drinking water supplemented with sucrose (15 g/L) for 48 hours to avoid early mortality due to excessive insulin released from damaged pancreatic beta cells [30]. Blood samples from the tail vein were used to monitor blood glucose levels 48 hours post injection using a glucometer (Optimum Exceed, Abbott Diabetes Care Inc. Alameda, CA, USA). Rats were considered diabetic when their glycemia exceeded 11 mmol/L (1mmol = 18mg glucose)[31]. The diabetic rats were divided into 2 groups- diabetes mellitus (DM, n = 10) and diabetes mellitus with insulin therapy (DM + INS, n = 6). The insulin-treated rats were given 5U/day of 24h-acting insulin (Human long acting Insulin, Eli Lilly & Company, IN) subcutaneously [32] beginning from 3rd day after induction of diabetes till the end of the study. Blood glucose was monitored daily. For baseline serum analysis, blood collection was performed under general anesthesia, i.e, Ketamine 75mg/kg and Xylazine 10mg/kg (intraperitonial injection, Sigma Chemical Co., St. Louis, MO, USA), through retro-orbital bleeding. For exosome enrichment, 18-hour urine samples were collected using metabolic cages (Lab Products, USA) before diabetes induction and at the 3^th^, 6^th^ and 9^th^ week following diabetes induction. Rats were euthanized at the 10^th^ week under Isoflurane 2% anesthesia (Sigma Chemical Co., St. Louis, MO, USA). Blood collection was performed through cardiac puncture. Kidneys were collected after perfusion with 1X phosphate-buffered saline (PBS). Left kidneys were kept in 4% paraformaldehyde for histological analysis whereas right kidneys were stored at -80°C for RNA isolation. The insulin levels in serum were analyzed using a RAT INSULIN EIA KIT (SPI Bio, Bertin Group, Montigny Le Bretonneux, France). All efforts were made to minimize animal suffering. For **study 2**, rats were treated and divided as above into three groups DM, CTRL and DM + INS (n = 9/group). Rats were euthanized as described above at weeks 3, 6 and 9 of the study (n = 3/time point/group) and their kidneys were preserved for histopathological analysis.

### Urine analysis

Urine albumin was estimated using a rat albumin ELISA kit (Bethyl Laboratories, TX, USA). Urine creatinine was analyzed using a modified Jaffe’s method (Randox, Crumlin County Antrium, UK).

### Enrichment of urinary exosomes

Exosomes were enriched by differential centrifugation of urine samples as described previously [25]. For electron microscopy, exosomal pellets were resuspended in 1X PBS.

### Electron microscopy of urinary exosomes

Urinary exosomal vesicle suspension in 1X PBS was applied to 300 mesh carbon coated copper grids (Ted Pella Inc., CA, USA). The adsorbed exosomes were negatively stained with 1% aqueous uranyl acetate. The samples were examined with a JEM 2100F electron microscope (JEOL, Peabody, MA, USA) operating at 200 kV.

### RNA isolation

Total RNA was extracted from the rat urinary exosomal pellet using miRNeasy Mini kit (Qiagen, Valencia, CA) as per manufacturer's instructions. Total RNA from rat kidney cortex was extracted using RiboZol Reagent (Amresco, Ohio, USA).

### Library preparation

Deep sequencing of small RNA was performed at Genotypic Technology Pvt. Ltd. (Bengaluru, India). For sequencing, equal amounts of urinary exosomal RNA (ng) were pooled from 3 rats at two—time points (before diabetes induction and at the 9^th^ week post-diabetes induction). About 200 ng of pooled RNA sample, enriched for small RNA, was used for small RNA library preparation according to standard protocol of TruSeq Small RNA Sample Prep Kit (Illumina, San Diego, CA, USA). The library was size selected in the range of 140–160 bp followed by overnight gel elution and salt precipitation. The prepared library was quantified using a Qubit Fluorometer (Life Technologies, NY, USA), and validated for quality on a High Sensitivity Bioanalyzer Chip (Agilent Technologies, Santa Clara, USA).

### Deep sequencing and analysis

Sequencing was done using Illumina HiSeq (Illumina San Diego, CA, USA) platform as per manufacturer’s instructions. Sequences were size sorted and reads having length between 16–36 bases was used for further analysis after adaptor and low quality sequence removal. Redundant reads having multiple hits at different genomic locations were removed from the dataset for downstream data analysis. The entire RNAseq dataset was mapped to Rat Genome Assembly Version 6.0 (Rnor_6.0) from EBI using bowtie 2.2.6 [33]. We downloaded the gff (genome feature file) from mirBASE 21 that is currently available for Rnor_5.0 (Assembly version 5.0). We mapped these miRNA back to Rat Genome Assembly Version 6.0 to generate gff files on Rnor_6.0. The mapped RNAseq data to Rnor_6.0 was overlapped with mirBASE 21[34] gff file for assigning functions to the RNAs. miREAP version 0.2 was used for predicting miRNA from Rnor_6.0. The mapped data was merged with predicted miRNA dataset to make novel miRNA annotated gff (genome feature file). This file was merged with EBI reference annotation file for Rnor_6.0 for making the final annotation data file for downstream data analysis [S1 File].

### Quantitative Real-time PCR

For microRNA expression analysis, cDNA was prepared from 10 ng total RNA (from UE and kidney tissue) using TaqMan microRNA Reverse Transcription Kit according to manufacturer's instructions (Applied BioSystems, Foster city, CA, USA) through reverse transcription PCR. Relative expression of miR-451-5p and miR-16 was estimated using TaqMan microRNA expression assays (Applied BioSystems, Foster city, CA, USA) through quantitative Real Time PCR (qPCR). Data were analyzed following the 2^-ΔCt^ method, using U6 snRNA as an endogenous control to normalize any input and cDNA conversion efficiency variations.

To analyze the expression of target genes of miR-451-5p and miR-16, first the kidney tissue total RNA (2μg) was reverse transcribed to cDNA using High-Capacity cDNA Reverse Transcription Kit as per manufacturer's instructions using random primers (Applied BioSystems, Foster city, CA, USA). The cDNA was diluted 2 times prior to use for qPCR. To estimate the relative gene expression of targets IL-6 and MMP-9, qPCR was performed using SYBR Green chemistry with GAPDH as endogenous control in kidney tissue as described previously using the 2^-ΔΔCt^ method [35, 36]. All qPCR reactions were performed in triplicates. The sequence of the primers used were as follows: For MMP-9 5’- ATGGTTTCTGCCCCAGTGAG -3’ and 5’- CCTTTAGTGGTGCAGGCAGA -3’; For GAPDH 5’-AGGTCGGTGTGAACGGATTTG-3’ and 5’-TGTAGACCATGTAGTTGAGGTCA-3’; For IL-6, 5’- CCCAACTTCCAATGCTCTCCT -3’ and 5’- GGATGGTCTTGGTCCTTAGCC -3’.

### Immunoblotting

Western blotting of exosomal proteins were performed as described by us previously [25, 37]. Briefly exosomal protein samples were solubilized in Laemmli sample buffer and equal volume of solubilized protein (12 μl) were loaded for each sample onto 10% polyacrylamide gel. Separated proteins were transferred onto nitro-cellulose membranes and blocked with 5% non-fat dry milk for 1 hr. Membranes were then incubated with primary antibody of exosomal marker mouse monoclonal TSG101 (Abcam, MA, USA) overnight at 4°C. After the incubation with primary antibody membranes were washed and then incubated with horse radish peroxidase conjugated appropriate secondary antibody (1:5000). The antibody-antigen reactions were visualized by using chemiluminescence (GE Healthcare, NJ, USA).

### Kidney tissue histology

Kidney tissues, fixed in 4% paraformaldehyde were processed in paraffin, sectioned at 3μm and stained with Periodic acid-Schiff (PAS) and Masson Trichrome (MT) according to the manufacturer’s instructions (Sigma, St Louis, MO) [38]. Stained sections were used for the following analysis using a Nikon Eclipse 80i light microscope. PAS-stained sections were examined for the degree of glomerular damage (*Glomerulosclerotic Index****)*** and MT stained sections were examined for the degree of tubulointerstitial fibrosis using a semiquantitative scoring method as described by Maric et al. 2004 [39]. Diabetic rats' kidney sections (from DM and DM + INS) were compared with kidney sections from CTRL rats.

#### Glomerulosclerotic index

PAS-stained sections were examined for the degree of glomerular damage (*Glomerulosclerotic Index)* using a semiquantitative scoring method as described by Maric et al. 2004 [39]. Briefly, the glomeruli were graded as; grade 0, normal glomeruli; grade 1, sclerotic area up to 25% (minimal sclerosis); grade 2, sclerotic area 25 to 50% (moderate sclerosis); grade 3, sclerotic area 50 to 75% (moderate-severe sclerosis); grade 4, sclerotic area 75 to 100% (severe sclerosis). The glomerulosclerotic index GI score was calculated using the following formula: GI score = (1xn1) + (2 x n2) + (3 xn3) +(4 x n4)/n0 +n1 +n2 +n3 +n4, where nx is the number of glomeruli in each grade of glomerulosclerosis. This analysis was performed with the observer masked to the treatment. One hundred glomeruli per section were analyzed.

#### Assessment of tubulointerstitial fibrosis

MT stained sections were examined for the degree of tubulointerstitial fibrosis using a semiquantitative scoring method as described by Maric et al. 2004 [39]. The degree of tubulointerstitial fibrosis was graded on a scale of 0 to 4: grade 0, affected area 0% (normal); grade 1, affected area less than 10%; grade 2, affected area 10 to 25%; grade 3, affected area 25 to 75%; grade 4, affected area greater than 75%. Estimation of tubulointerstitial fibrosis was performed with the observer masked to the treatment groups.

### Statistical analysis

Quantitative data are expressed as mean ± SEM. Comparisons within groups were made using paired Student t-tests. One-way ANOVA followed by a multiple comparisons testing was used to assess differences between individual pairs of means among the groups. P values < 0.05 were considered significant for all tests using Sigma Plot 12.3 (Chicago, IL). Fold change in expression by RT-PCR were calculated by 2^-ΔCt^ method, where ΔCt = Ct_gene of interest_−Ct _endogenous control_. The 2^-ΔCt^ values were log transformed for statistical analyses and representation. Pearson Correlation test was used for correlation analysis. Differential expression analysis was carried out using 2 different approaches. The Tuxedo analysis suite [33, 40] was used for transcript assembly as well as for differential expression analysis [S1 File]. We used DESEQ2 tool [41] to compute differential expression in both the samples at the non-assembled transcript level., The expression (read count) of each miRNA in a sample was normalized by a scaling factor calculated by DESeq2. (DESeq2 manual link for detail: https://www.bioconductor.org/packages/3.3/bioc/vignettes/DESeq2/inst/doc/DESeq2.pdf). Bowtie was used for mapping clean raw reads into the Rnor_6.0 and htseq was used for preparing input data files for DESEQ2. Mapping statistics is presented in S1 File. We used exon level mapping data count as the input for DESEQ2. Target region coverage is given in S1 File.

## Results

### Diabetes induction and characterization of urinary exosomes

**Fig 1:**
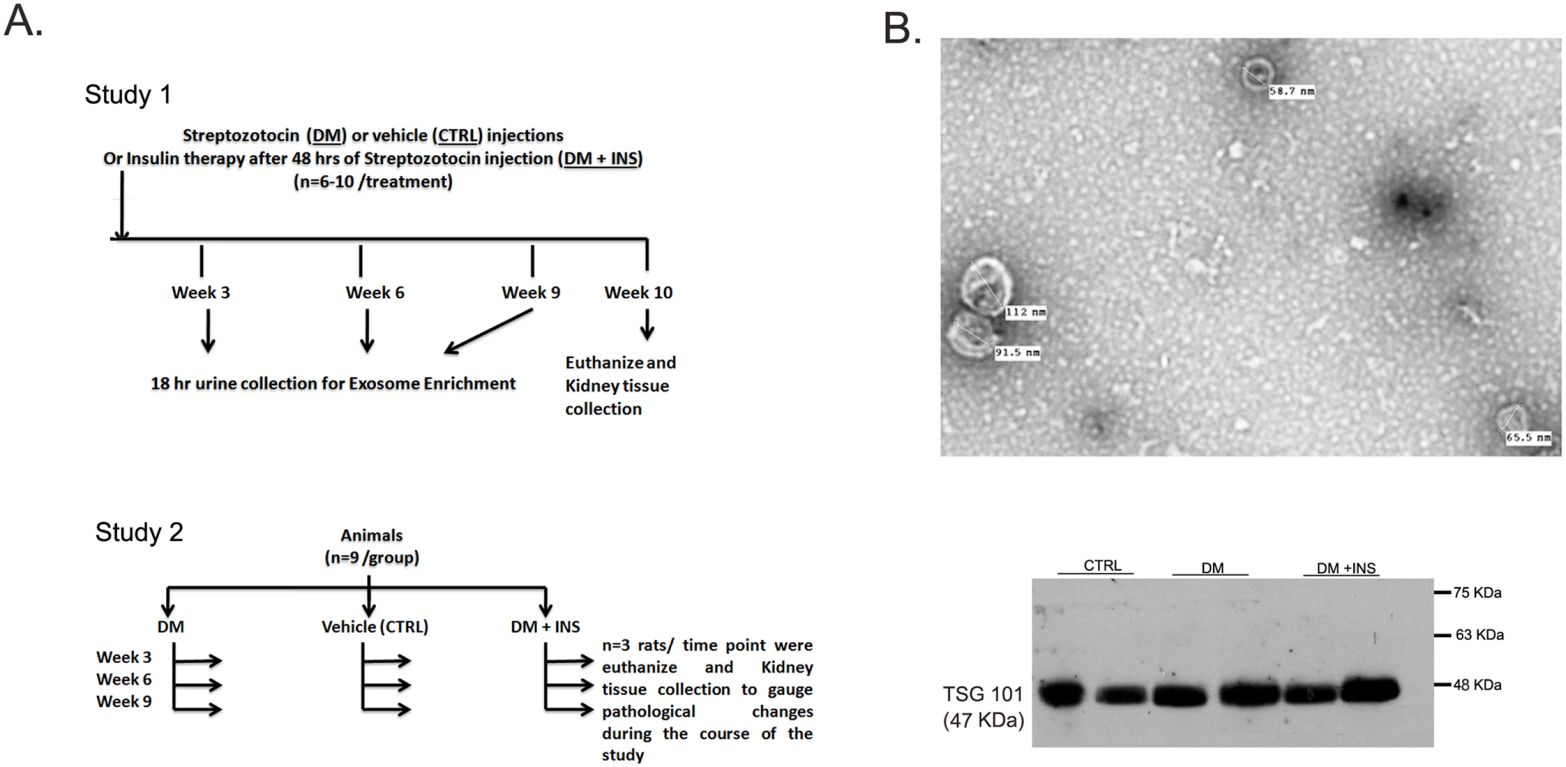
Study protocols and validation of rat urinary exosomes. (A) Schematic of the experimental protocols, (B) Representative electron microscope images of exosomes vesicles isolated from 18 hours rat urine at X10,000 magnification. The images shows small, round vesicles of size between 30-120nm. Below is the representative immunoblots for TSG101 proteins in urinary exosome samples from, untreated diabetic (DM), non-diabetic control (CTRL) and insulin treated diabetic rats (DM + INS). Exosome pellet from urine samples from all rats were positive for TSG101 protein, an exosomal marker protein.

Fig 1A is a schematic representation of study protocols. The exosomes enriched from urine samples from the rats were examined by transmission electron microscopy using negative staining procedure with 1% aqueous uranyl acetate. Electron micrographs showed the presence of small, round vesicles of size 30-120nm (Fig 1B). The enrichment of exosomes was further confirmed by the presence of a specific band for TSG101, an exosomal marker protein, in the exosomal protein samples by immunoblotting (Fig 1B).

**Fig 2:**
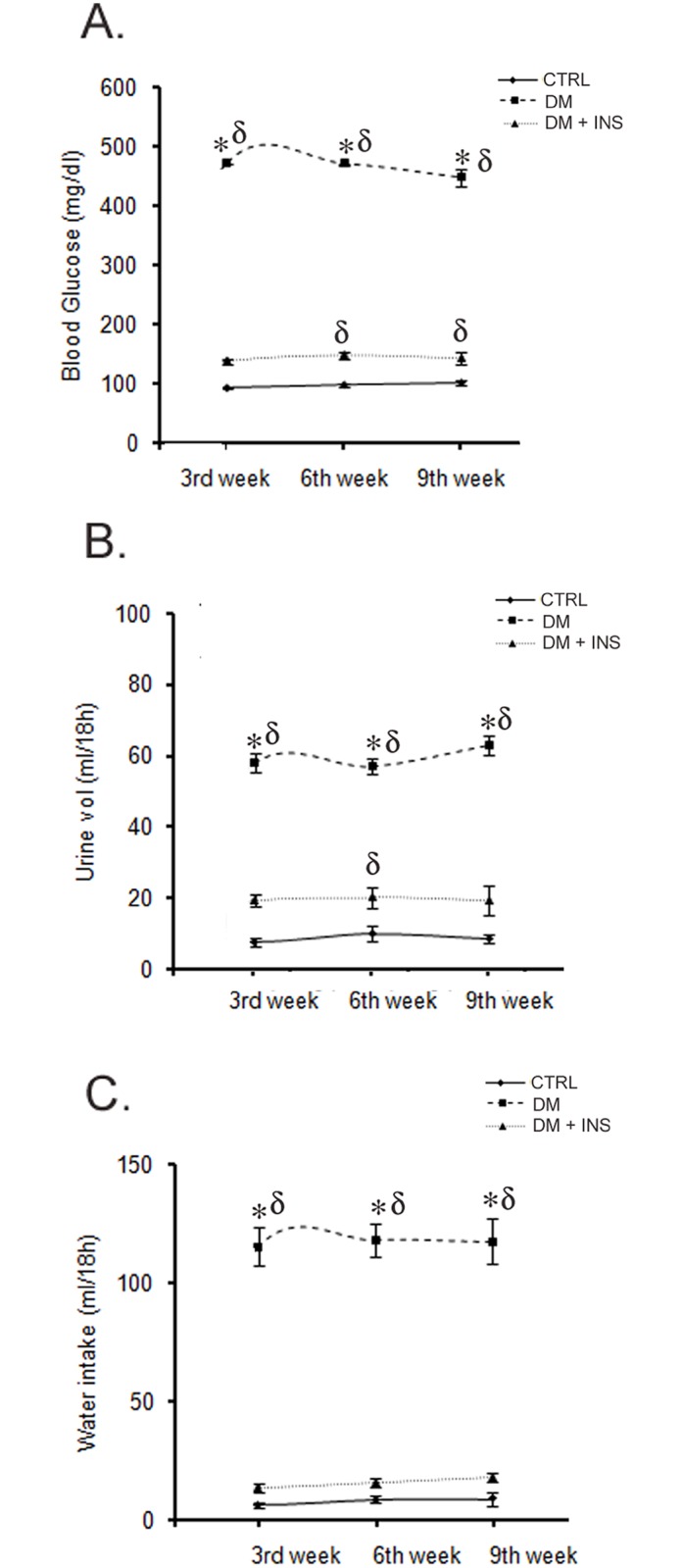
Validation of diabetes induction in rats. (A) Line diagram showing mean blood glucose, and (B-C) 18-hour urine volume and water intake in untreated diabetic (DM, n = 10), non-diabetic control (CTRL, n = 6) and insulin treated diabetic rats (DM + INS, n = 6) at following weeks during the course of the study 3rd, 6th and 9th weeks post-injections *p≤0.05 versus DM+ INS and δ p≤0.05 versus CTRL by One-Way ANOVA followed by pair wise multiple comparison (at each time point) testing (n = 6-10/group).

Diabetes induction was validated by analyzing blood glucose, 18-hour urine volume, and water intake. All these parameters were significantly higher in DM rats relative to CTRL or DM + INS groups during the entire course of study (p<0.001, Fig 2). Moreover, serum insulin levels in DM rats were (ng/ml): 4.2 ± 0.3 and 0.09 ± 0.03 (p<0.001) before and 9 weeks after diabetes induction, respectively.

### Change in renal pathology and albumin excretion during the course of the study

**Fig 3:**
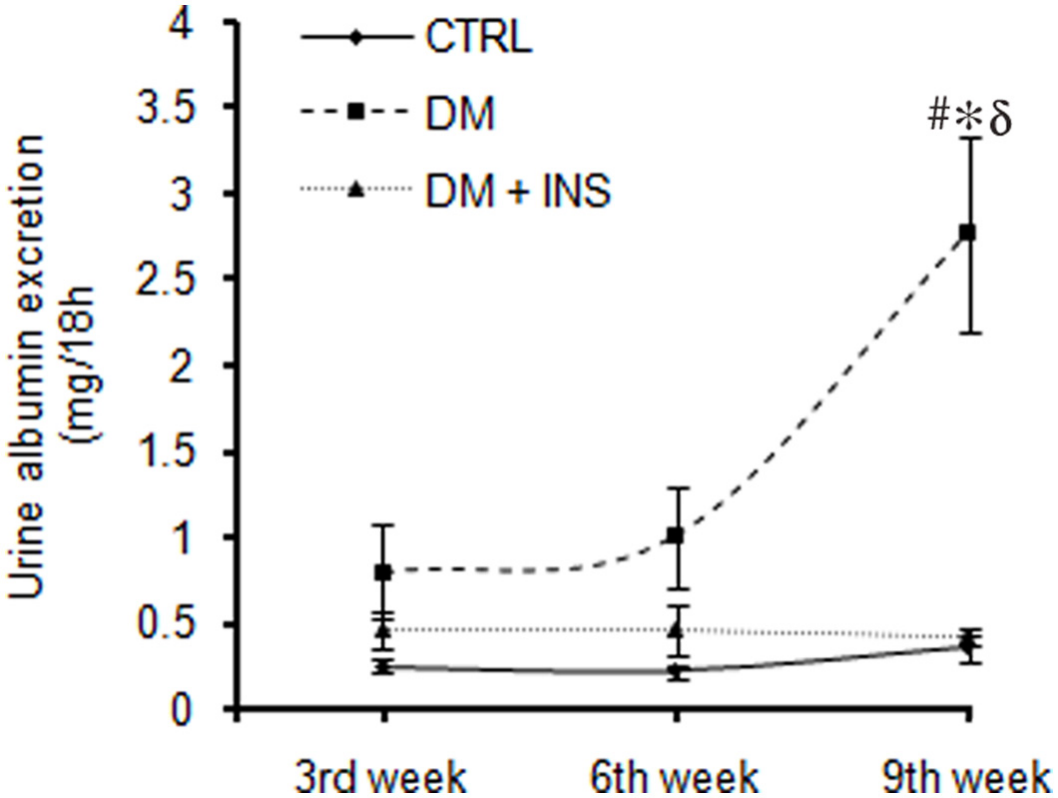
Urine albumin excretion in rats. Line plot showing average urine albumin excretion (mg of albumin in 18 hours urine collected from untreated diabetic (DM, n = 10), non-diabetic control (CTRL, n = 6) and insulin treated diabetic rats (DM + INS, n = 6) at following weeks during the course of the study (3^rd^, 6^th^ and 9^th^ weeks post-injections. #p ≤ 0.05 as compared to 3^rd^ week by unpaired t-test (n = 10/time point). *p≤0.05 versus DM+ INS and δ p≤0.05 versus CTRL by One-Way ANOVA followed by pair wise multiple comparison (at each time point) testing (n = 6-10/group).

Although urine albumin was modestly elevated in DM (over CTRL and DM + INS groups) at 3 and 6 weeks, a marked rise was not noted until 9-weeks (Fig 3). Mean albumin excretions were (mg/18-hours) 0.79 ± 0.27, 1.0 ± 0.29 and 2.75 ± 0.56, respectively for weeks 3, 6 and 9. Moreover, a significant rise in urine albumin could not be detected between 3 and 6 weeks (p = 0.1). No significant change in urine albumin excretion was observed in CTRL or DM + INS rats during the entire course of study, n = 6/group, Fig 3.

**Fig 4:**
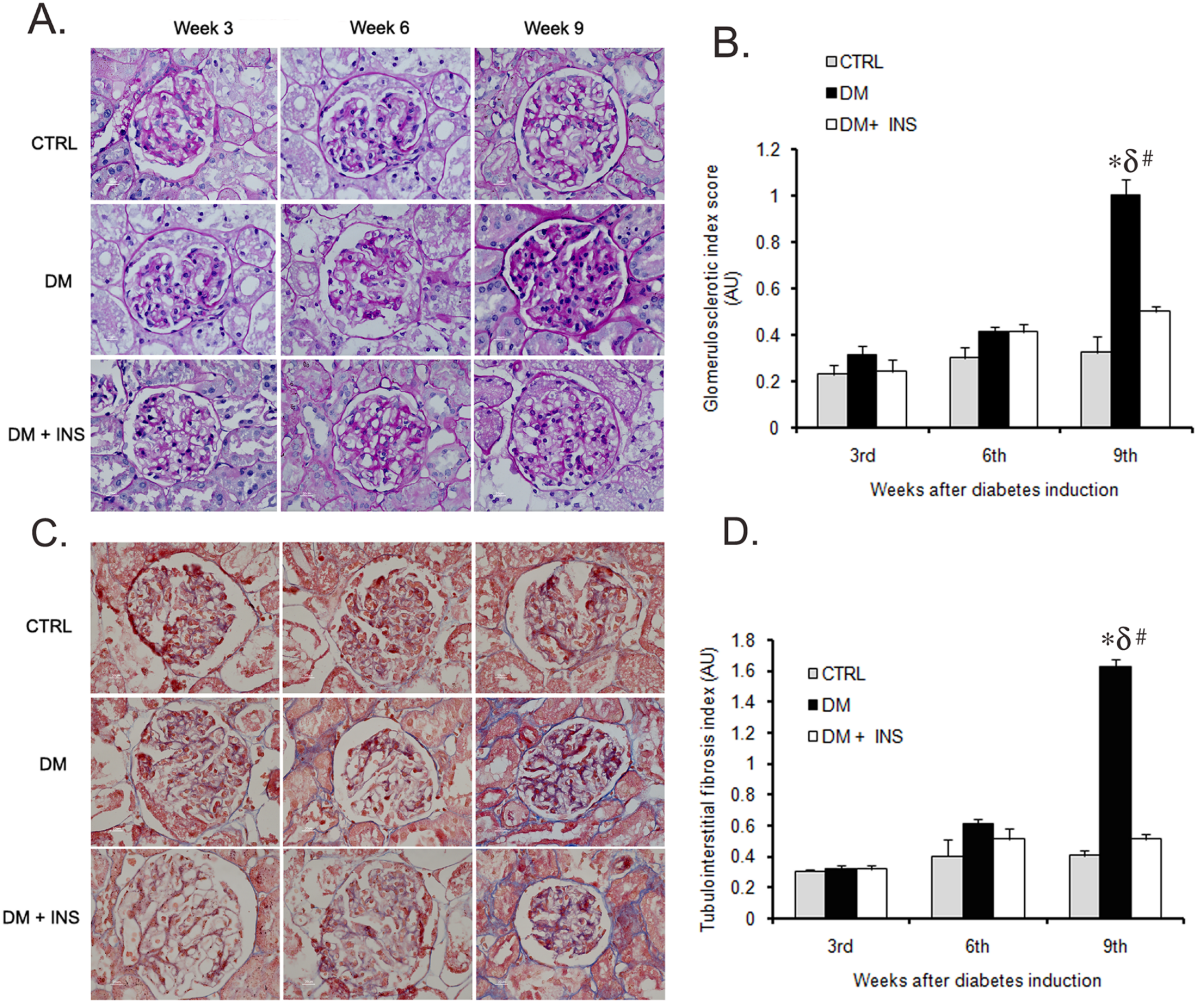
Kidney tissue pathology in rats. Representative (A) PAS and (C) MT stained images of kidney tissue sections from untreated diabetic (DM), non-diabetic control (CTRL) and insulin treated diabetic rats (DM + INS) after 3^rd^, 6^th^ and 9^th^ weeks of diabetes induction (n = 3/group/time point), Bar graph showing a semi-quantitative analysis for (B) glomerulosclerotic index (GI) and (D) tubulointerstitial fibrosis index (TFI) kidneys tissues from these (n = 3/group/time point). #p≤0.05 as compared to 3^rd^ week within the group by unpaired t-test, (n = 3/time point). *p≤0.05 versus DM+ INS and δ p≤0.05 versus CTRL by One-Way ANOVA followed by pair wise multiple comparison (at each time point) testing (n = 3/group).

Renal pathology during the progression of diabetes was assessed by PAS and MT staining of kidney tissue from Study-2 rats. Results revealed intense staining for collagen and glycogen deposition in kidney sections at the 9^th^ week of diabetes, as compared to kidney tissue sections from the 3^rd^ and 6^th^ weeks of diabetes. However, no significant differences in staining intensity were observed at the 3^rd^ and 6^th^ weeks of the study between any of the groups (Fig 4A and 4C). Furthermore, semi-quantitative analysis of PAS- and MT-stained kidney tissue exhibited significantly higher glomerulosclerosis index (GI) and tubulointerstitial fibrosis index (TFI) scores, respectively only in kidneys from the DM group at week 9, relative to kidney sections at week 3 or week 6 (Fig 4B and 4D). Kidney tissue sections from rats in the CTRL or DM + INS groups showed no significant differences during the course of the study (n = 3/time point/group, Fig 4)

### Change in urinary exosomal miRNA-451-5p and miR-16 levels during the course of diabetes

A pilot sequencing experiment was performed using pooled RNA samples from 3 diabetic rats for identification of candidate miRNA regulated at 9th week of diabetes compared to their non-diabetic state (data not shown). Since molecules showing “increased” levels in pathological conditions are generally considered to be the better biomarkers, in a pilot study, we validated the expression levels of up-regulated micro-RNAs by qPCR. The results from miR-451-5p and miR-16 corroborated with the findings from deep sequencing analysis most, and hence they were selected for further analysis.

**Fig 5:**
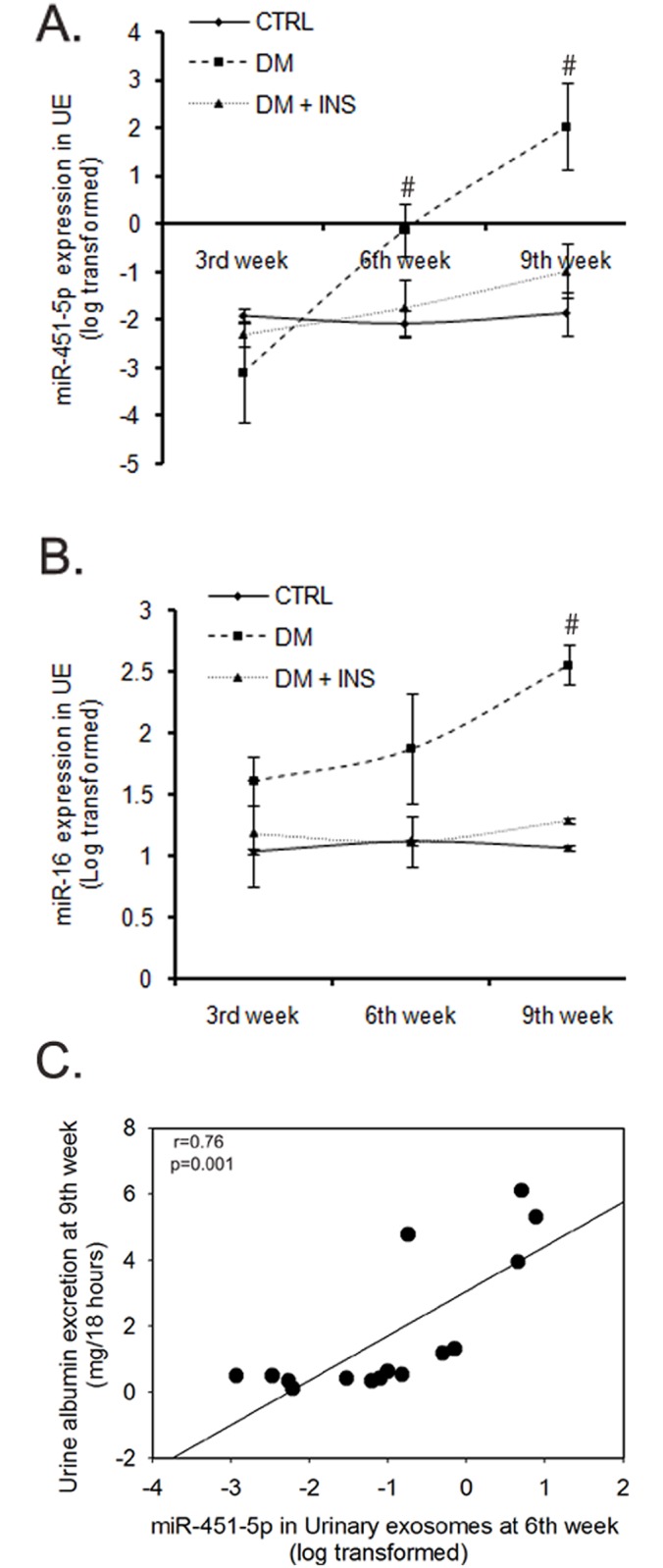
Change in Urinary exosomal miRNA-451-5p and miR-16 levels during the course of the study in rats. (A) Line diagram showing average fold expression of (A) miR-451-5p and (B) miR-16 in urinary exosomes from untreated diabetic (DM, n = 10), non-diabetic control (CTRL, n = 6) and insulin treated diabetic rats (DM + INS, n = 6) at 3^rd^, 6^th^ and 9^th^ weeks post-injection. Fold expression was calculated using the 2^- ΔCT^ method, where ΔCT = CT_miRNA_—CT_U6snRNA_. The values were log transformed for analysis. #p≤0.05 versus week 3 by paired t-test, (n = 6-10/time point). (C) Scatter plot with regression fit line showing the relationship between miR-451-5p at 6^th^ week and urine albumin at 9^th^ week.

To determine the time course of regulation of miR-451-5p and miR-16 in urinary exosomes during diabetes, we examined UE from rats at the 3^rd^, 6^th^ and 9^th^ weeks of diabetes by Taqman-based qPCR. The results revealed a significant rise in the levels of both of these miRNAs during the progression of diabetes. For miR-451-5p, the levels increased significantly in the 6^th^, and then further increased in the 9^th^ week of diabetes, relative to the 3^rd^ week (p = 0.03 and p = 0.002, respectively Fig 5A). For miR-16, however, a significant rise was observed only in the 9^th^ week of diabetes, relative to the 3^rd^ week (p = 0.03, Fig 5B and S3 Table). Moreover, UE miR-451-5p levels at 6^th^ week significantly correlated with urine albumin excretion at 9^th^ week (Fig 5C)

Rats in CTRL or DM + INS groups did not show any significant change in the levels of these miRNA during the course of the study (n = 6/group, Fig 5 and S3 Table). Moreover, at the 9^th^ week, a significant difference was observed for miR-451-5p (p = 0.04) and miR-16 (p = 0.03) among the three groups by One Way ANOVA.

### Kidney tissue levels of miR-451-5p and miR-16 and their association with renal pathology at the end of the study

**Fig 6:**
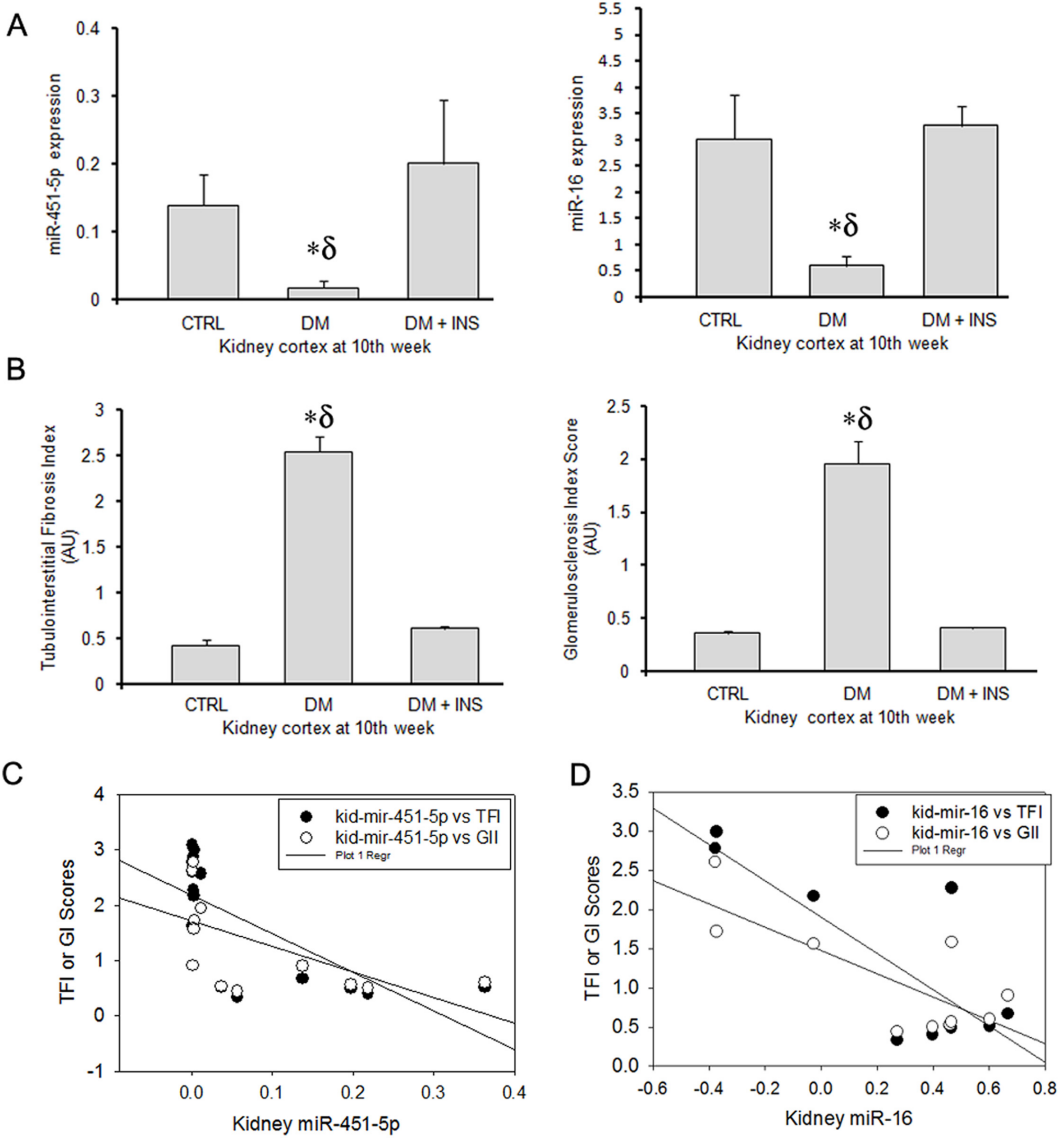
Kidney tissue levels of miR-451-5p and miR-16 and their association with renal pathology at the end of the study. Bar graphs showing (A) average fold expression of miR-451-5p and miR-16expression, fold expression was calculated using the 2^- ΔCT^ method, where ΔCT = CT_miRNA_—CT_U6snRNA_; and (B) semi-quantitative analysis of pathology for glomerulosclerotic index (GI) and tubulointerstitial fibrosis index (TFI) in kidney-tissue from untreated diabetic (DM), non-diabetic control (CTRL) and insulin treated diabetic rats (DM + INS) at 10^th^ week of the study 1 (n = 6-10/group). (C) Scatter plot with regression fit line showing the relationship between miR-451-5p and miR-16 expression with TFI and GI scores. *p≤0.05 versus DM+ INS and δ p≤0.05 versus CTRL by One-Way ANOVA followed by pair wise multiple comparison testing (n = 6-10/time point).

**Fig 7:**
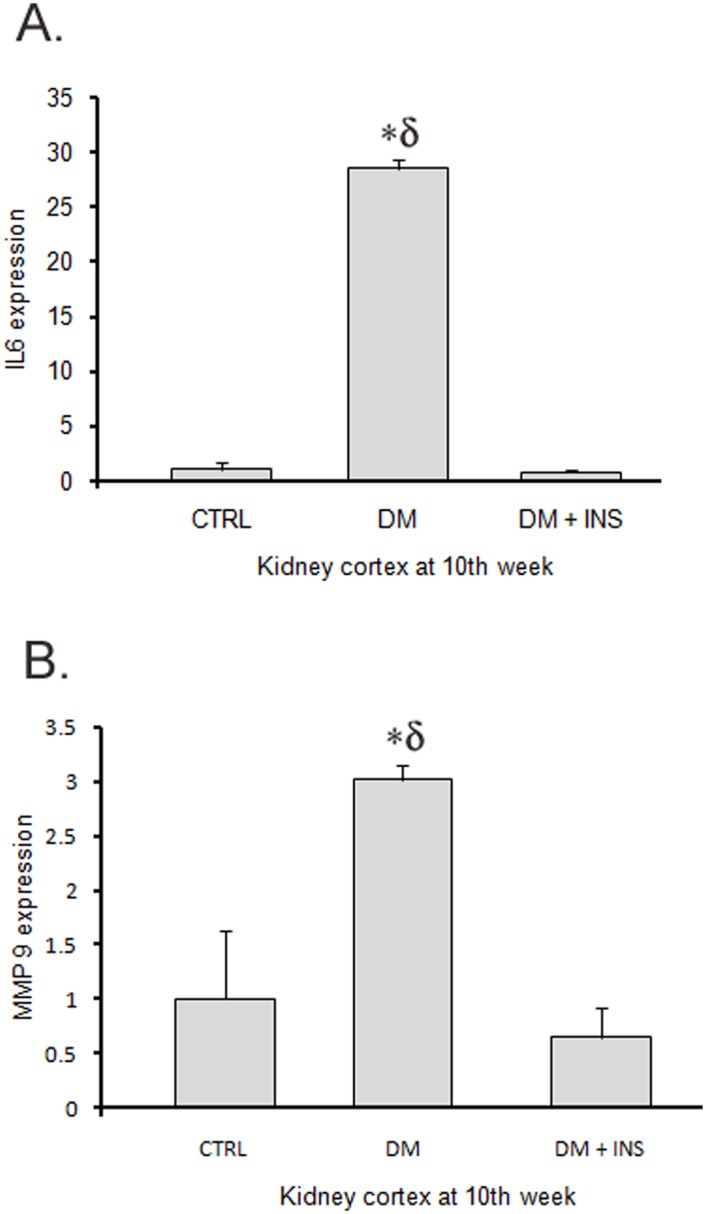
Kidney tissue levels of IL-6 and MMP-9 in rats. Bar graphs showing average fold expression of (A) IL-6 and (B) MMP-9 in kidney-tissue from untreated diabetic (DM), non-diabetic control (CTRL) and insulin treated diabetic rats (DM + INS) at 10^th^ week of the study 1 (n = 6-10/group). *p≤0.05 versus DM+ INS and δ p≤0.05 versus CTRL by One-Way ANOVA followed by pair wise multiple comparison testing (n = 6-10/time point).

We examined the effect of diabetes on the regulation of miRNA-451-5p and miR-16 in the kidney tissue at 10 weeks (Study 1) by qPCR. Surprisingly, we found significantly lower levels of both miR-451-5p and miR-16 in kidney tissue from DM rats relative to control or DM + INS rats (p = 0.007 and 0.024, respectively by One-Way ANOVA, Fig 6A). As expected, tubulointerstitial fibrosis index (TFI) and glomerulosclerosis index (GI) scores were significantly higher in the kidney tissues from DM rats compared to CTRL or DM + INS (n = 6–10, p≤ 0.05, Fig 6B). However, renal pathology showed a strong negative association with the renal expression of miRs, i.e., for miR-451-5p, n = 14, r = 0.70, p = 0.005 for TFI, and r = 0.6 and p = 0.02 for GI, and for miR-16, n = 10, r = 0.8, p = 0.005 for TFI and r = 0.78, p = 0.008 for GI (Fig 6C). Significantly higher mRNA levels of IL-6 and MMP-9 genes (predicted targets for miR-16 and miR-451-5p) were found in kidney tissue from DM rats relative to control or DM + INS rats (Fig 7).

## Discussion

Analysis of miRNA excretion during diabetes progression holds promise as an adjunct in disease management, due to its potential to predict the earliest stages of nephropathy [42–44]. Findings presented in our study on the regulation of miR-451-5p support the potential usefulness of urinary exosomal (UE) miRNA analysis for non-invasive serial monitoring of early renal damage in diabetes. Electron micrographs and immunoblotting with an exosomal marker, TSG 101 confirmed the presence of exosomes collected via high-speed centrifugation from rat urine samples. However, the source of urinary exosomes containing altered miRNA cannot be confirmed by our study. It is possible that exosomes released from various tissues, including heart, may be filtered by the kidney from blood, and end up in the urine [45, 46]. Moreover, it is unclear to what extent changes in the levels of miR-16 and miR-451-5p in urinary exosomes in diabetes can predict future renal pathology.

Notably, mean miR-451-5p levels increased more than 1000-fold between 3 and 6 weeks, while urine albumin increased only 21% (non-significant). Furthermore, significant renal pathology was not observed with diabetes at this time point (Fig 4). Thus, the rise in miR-451-5p in UE at the 6^th^ week (or even earlier if sampled) may predict an increase in urine albumin excretion and renal tissue pathology. This is further supported by a strong positive correlation of urine albumin at 9 weeks with UE miR-451-p levels at 6^th^ weeks in rats found in our study (Fig 5C). This may support its potential usefulness for predicting those subjects with diabetes susceptible to renal damage. However, further studies are warranted to establish this potential.

However, urine exosomal miRNA did not correlate or predict renal miRNA levels, at least with regard to miR-451-5p and miR-16. Both of these miRs had significantly lower expression in diabetic-rat kidneys at 10^th^ weeks of diabetes, compared to non-diabetic rats (Fig 6A). Moreover, lower kidney-tissues levels of these miRNAs, were associated with higher indices of renal pathology (GI and TFI scores, Fig 6C), supporting the possibility that these miRNAs have protective roles, perhaps via targeting a common set of fibrotic/inflammatory genes in the kidney tissue. This is further supported by significantly higher renal expression of interleukin-6 (IL-6), a marker of an inflammatory milieu and a common target predicted for these miRNAs (Fig 7). In this regard, a recent study by Liang et al, 2015 has shown that miR-16 targets the 3'-untranslated region of IL-6 and suppressed its translation in mesangial cells [47]. Furthermore, Rosenberger et al, 2015 have shown that the inhibition of miR-451 expression is related to elevated levels of IL-6 [48]. In addition the levels of MMP-9, experimentally proven target of miR-451 reported in mirTarbase was also significantly increased in diabetic kidney tissue (Fig 7). Nevertheless, both MMP-9 and IL-6 have been associated with pathogenesis of diabetic nephropathy [49–52].

It is unclear why renal levels of these two miRNAs are so markedly reduced in the diabetic rat kidneys. We speculate that during uncontrolled diabetic conditions more excretion of these miRNAs via urinary exosomes may have resulted in the drop in their kidney-tissue levels [53]. The drop in kidney levels of miR-451-5p and miR-16 could lead to unchecked increased expression of their target IL-6 and MMP-9 levels (Fig 7) which are associated with the pathogenesis of diabetes nephropathy [52] [49–51]. Thus, we hypothesize a protective role of these miRNAs in kidney tissue.

A similar possible explanation could be that as diabetic nephropathy progresses, kidney cells can no longer repair themselves, as effectively, and the expression of these miRNAs is reduced. Therefore, in rats that are adapting well, the expression of these miRNAs is higher than in the rats that are "decompensated" and have poor prognosis. This is in agreement with studies demonstrating a protective role of miR-451 in ameliorating diabetic complications, especially diabetic nephropathy [54, 55]. Moreover, the down-regulation of miR-451 has also been associated with cardiomyopathy in diabetic mice [46]. In addition to diabetes, its tumor suppressive and anti-proliferative potential has indicated its therapeutic role in cancers [56, 57].

In summary, we demonstrated a progressive rise in miR-451-5p levels and miR-16 in urinary exosomes in diabetic rats during the progression of diabetes. Both these miRNAs, were however, reduced in diabetic rats’ kidney and potentially have a protective role in kidney tissue. The rise in miR-451-5p may be a more sensitive predictor of imminent diabetic kidney disease, as compared to albumin excretion. Further studies are, however, warranted to establish the potential of miR-451-5p as a non-invasive tool to serially monitor diabetic renal damage in human subjects.

## Supporting Information

S1 File Additional procedures and information related to sequencing data analysis. (DOC)

S2 File NC3Rs ARRIVE Guidelines Checklist 2014. (DOC)

S1 Table List of 447 known rat miRNAs detected in urinary exosomes in the non-diabetic state. (XLSX)

S2 Table List of differentially regulated miRNAs with log_2_fold change. (XLSX)

S3 Table Fold change values for miR-451-5p and miR-16 obtained by real time PCR. (DOC)
