# Expression of Concern: Urinary Exosomal microRNA-451-5p Is a Potential Early Biomarker of Diabetic Nephropathy in Rats

**DOI:** 10.1371/journal.pone.0316405

**Published:** 2024-12-23

**Authors:** The PLOS ONE Editors

After this article [1] was published, concerns were raised that the Fig 4A CTRL week 6 panel and DM week 3 panel appear similar.

The corresponding author confirmed that the panels were duplicated inadvertently and supplied the original image data (S1 File), the data underlying the graphs in Fig 4 (S2 File), and a corrected figure.

In addition, contrary to the article’s data availability statement, the raw data underlying this study were not provided with the article. The corresponding author provided the individual-level data underlying the results presented in Figs 2–3 and Figs 4–7 (S3 File). The underlying data for Fig 1B and S3 Table are either incomplete or unavailable; available data for S3 Table are in S4 File, and an updated S3 Table using the available data can be found in S5 File.

During the editorial assessment of the underlying data additional concerns were raised. Specifically:

Unusual repeats were found in the individual-level data underlying the results presented in Figs 2, 3, and 5, and S3 Table.

The primers reported for MMP-9 and IL-6 qRT-PCR do not appear to span the exon-exon junctions.

The corresponding author stated that it is not uncommon for rats to present the same within or between parameter values for blood glucose, body weight, and urine protein excretion. They commented that blood glucose was measured by glucometer, water intake and urine volume were measured by recording the amount in the graduated drinking water bottle and Falcon tube attached to the metabolic cages, and the albumin excretion (Fig 3) and log 2-fold expression (Fig 5) values used in the figures were rounded to 2 decimal places.

A member of the *PLOS ONE* Editorial Board reviewed the concerns and the primers reported in the article. Regarding the primer concerns, they stated that the primers are not optimal and do not span exon-exon junctions, but the primers reported for MMP-9 and GAPDH recognize the corresponding mRNAs. However, the reported IL-6 primers do not appear to recognize any transcript. Furthermore, the board member commented that the methods and reagents reported are acceptable and in line with community standards, but some information is missing, for example, the amplification cycles with the melting temperatures.

The Editorial Board member was satisfied with the authors’ response regarding the repeated data on glucose levels, drinking water, and creatinine secretion, but stated that they were unable to comment on the concerns raised with the underlying qRT-PCR data as the authors provided processed data instead of the raw data set for editorial review.

Editorial assessment of the manual for the glucometer used in this study [2] suggests that the device cannot read results greater than 500, reporting a “HI” reading instead of an exact blood glucose level, which may provide an explanation for the observation that the value 500 appears to be overrepresented in the DM blood glucose group in Fig 2. In the absence of accurate blood glucose readings, readers should interpret the corresponding results with caution.

The *PLOS ONE* Editors issue this Expression of Concern to notify readers that the article’s data availability statement is incorrect, to provide the available data, and to inform readers that in the absence of the complete underlying data set, the concerns with this article cannot be fully resolved and the results should be interpreted with caution.

**Fig 4:**
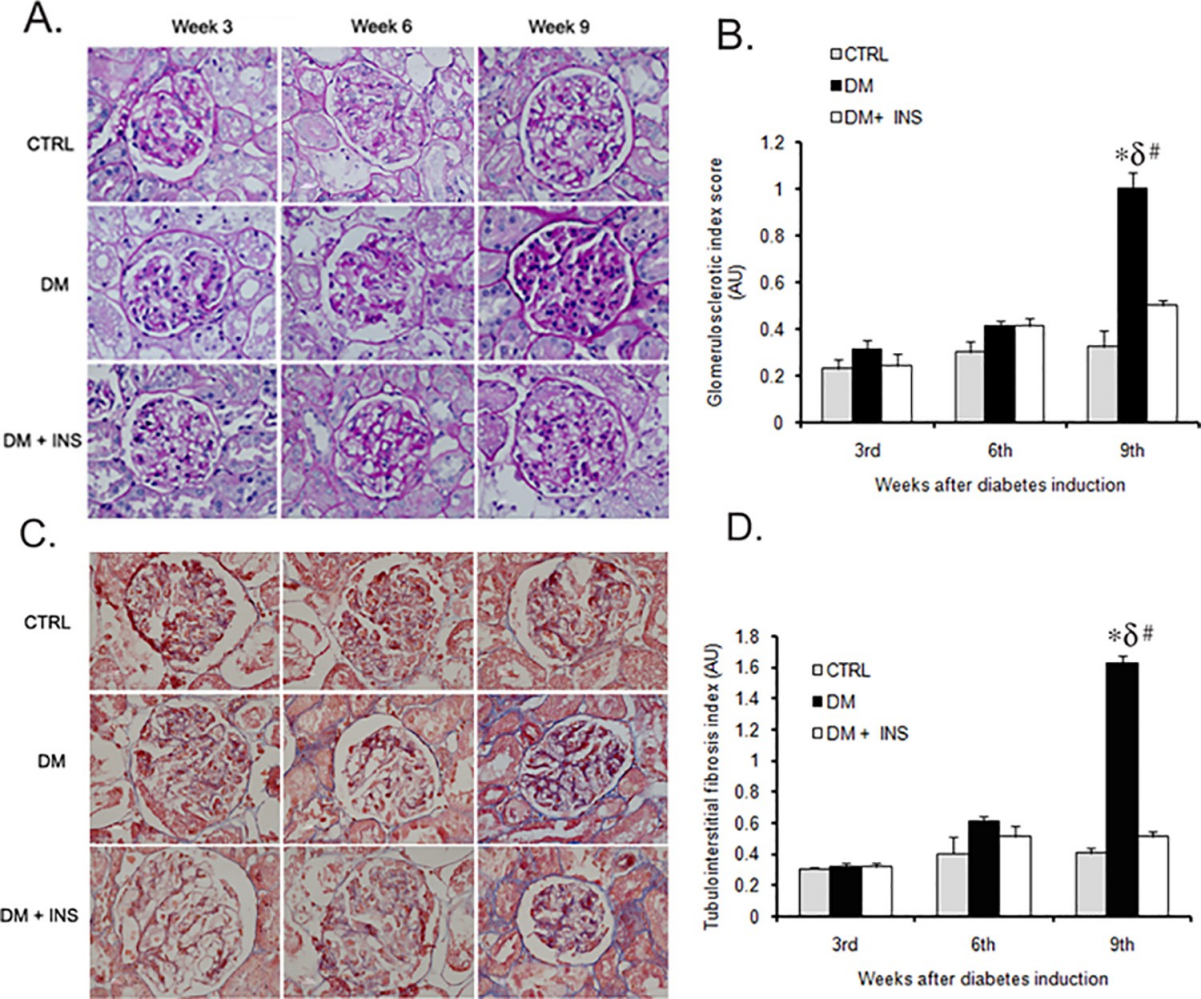
Kidney tissue pathology in rats. Representative (A) PAS and (C) MT stained images of kidney tissue sections from untreated diabetic (DM), non-diabetic control (CTRL) and insulin treated diabetic rats (DM + INS) after 3^rd^, 6^th^ and 9^th^ weeks of diabetes induction (n = 3/group/time point), Bar graph showing a semi-quantitative analysis for (B) glomerulosclerotic index (GI) and (D) tubulointerstitial fibrosis index (TFI) kidneys tissues from these (n = 3/group/time point). #p≤0.05 as compared to 3^rd^ week within the group by unpaired t-test, (n = 3/time point). *p≤0.05 versus DM+ INS and δ p≤0.05 versus CTRL by One-Way ANOVA followed by pair wise multiple comparison (at each time point) testing (n = 3/group).

## Supporting information

S1 File Original image data for Fig 4. (PPTX)

S2 File Data underlying graphs in Fig 4. (XLSX)

S3 File Data underlying graphs in Figs 2–3 and 5–7. (XLSX)

S4 File Fold expression data in S3 Table for Study 1 animals. (XLSX)

S5 File Updated S3 Table. (DOC)

## References

[1] Mohan A, Singh RS, Kumari M, Garg D, Upadhyay A, Ecelbarger CM, et al. (2016) Urinary Exosomal microRNA-451-5p Is a Potential Early Biomarker of Diabetic Nephropathy in Rats. PLoS ONE 11(4): e0154055. 10.1371/journal.pone.0154055 27101382 PMC4839711

[2] Abbott Optium Xceed Device Manual https://medaval.ie/docs/manuals/Abbott-Optium-Xceed-Manual.pdf

