# Supplementary material for: Urinary Exosomal microRNA-451-5p Is a Potential Early Biomarker of Diabetic Nephropathy in Rats
Source: PLoS One. 2016 Apr 21;11(4):e0154055. doi: 10.1371/journal.pone.0154055 (PMC4839711; doi:10.1371/journal.pone.0154055)

**Table 1: Mapping statistics of samples against R_nor6.0 Assembly using bowtie 2.2.6**

|  | **ND (Non-diabetic)** | **9th week** |
| --- | --- | --- |
| No. Of Features | 100599 | 133010 |
| Ambiguous | 3252 | 1622 |
| Too_low_quality | 400879 | 597344 |
| Not Aligned | 2421528 | 1709910 |

Table 2: Blast output of MiRBase21 against clean raw reads using 2 different blast parameters.

| **Cell type** | **Total number** | **No hits (plain blast) PB** | **No hits with short read flag on- SB** | **No. of sequences having hits -PB** | **No. of sequences having hits in SB**  **(>75% length at > 90% identity)** | **Unique miRNA in PB** | **Unique miRNA in SB** |
| --- | --- | --- | --- | --- | --- | --- | --- |
| ND  Non-diabetic | 2936925 | 2936521 | 1193082 | 149 | 24829 | 2 | 2238 |
| 9^th^ week | 2449156 | 2448839 | 1115450 | 177 | 23443 | 2 | 1749 |

Table 3: MiREAP output results

| **Cell Type** | **Total Number predicted by mireap (reads)** | **Uniq number predicted by mireap (uniq reads)** |
| --- | --- | --- |
| ND (Non-diabetic) | 2728 | 2192 |
| 9^th^ week | 3291 | 2660 |

**Figure 1: Data analysis flow chart for generating final annotation data file**

**
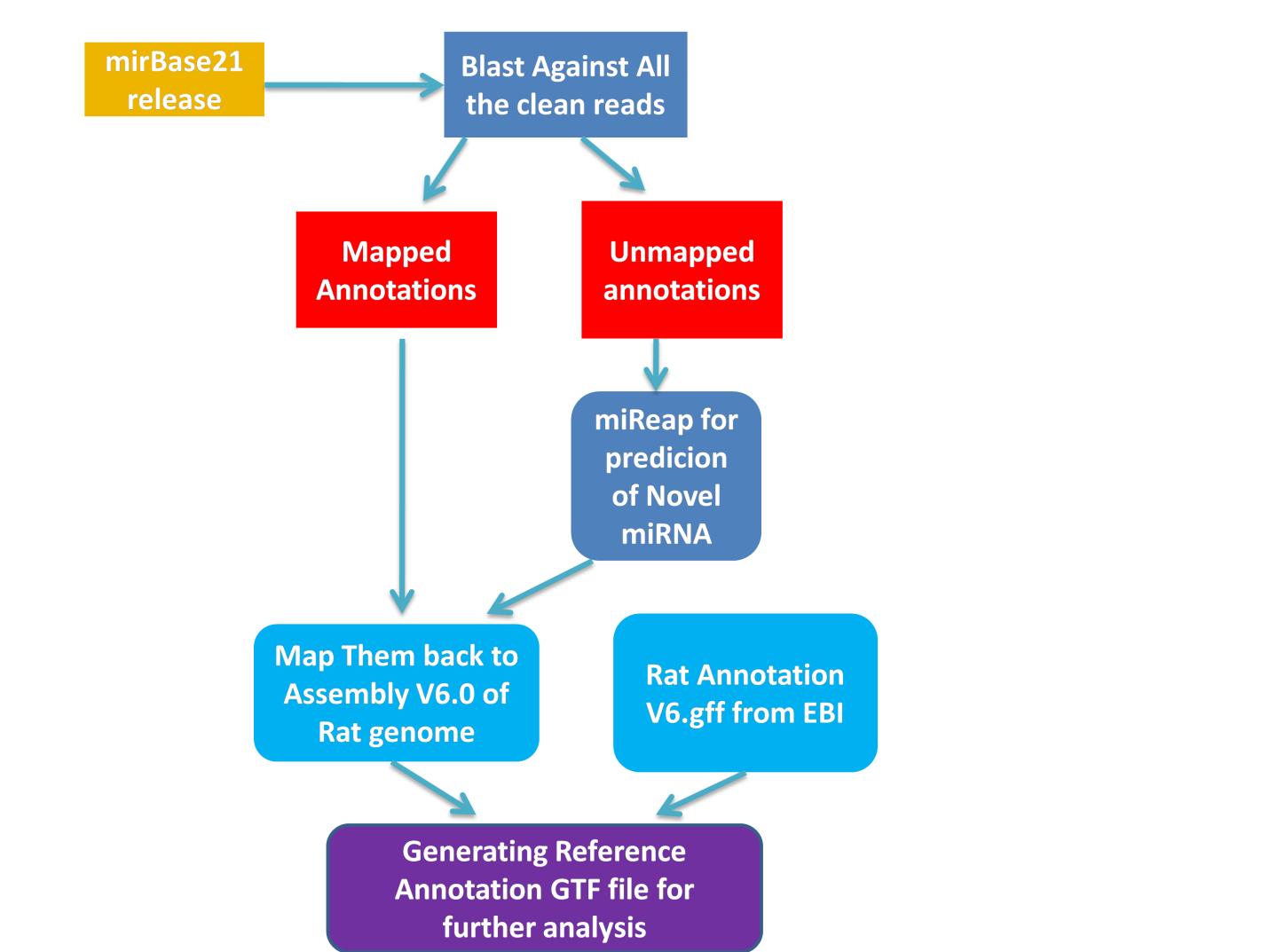
**

**Figure 2: Data analysis flow chart for transcript assembly and differential expression analysis using Tuxedo pipeline**

**
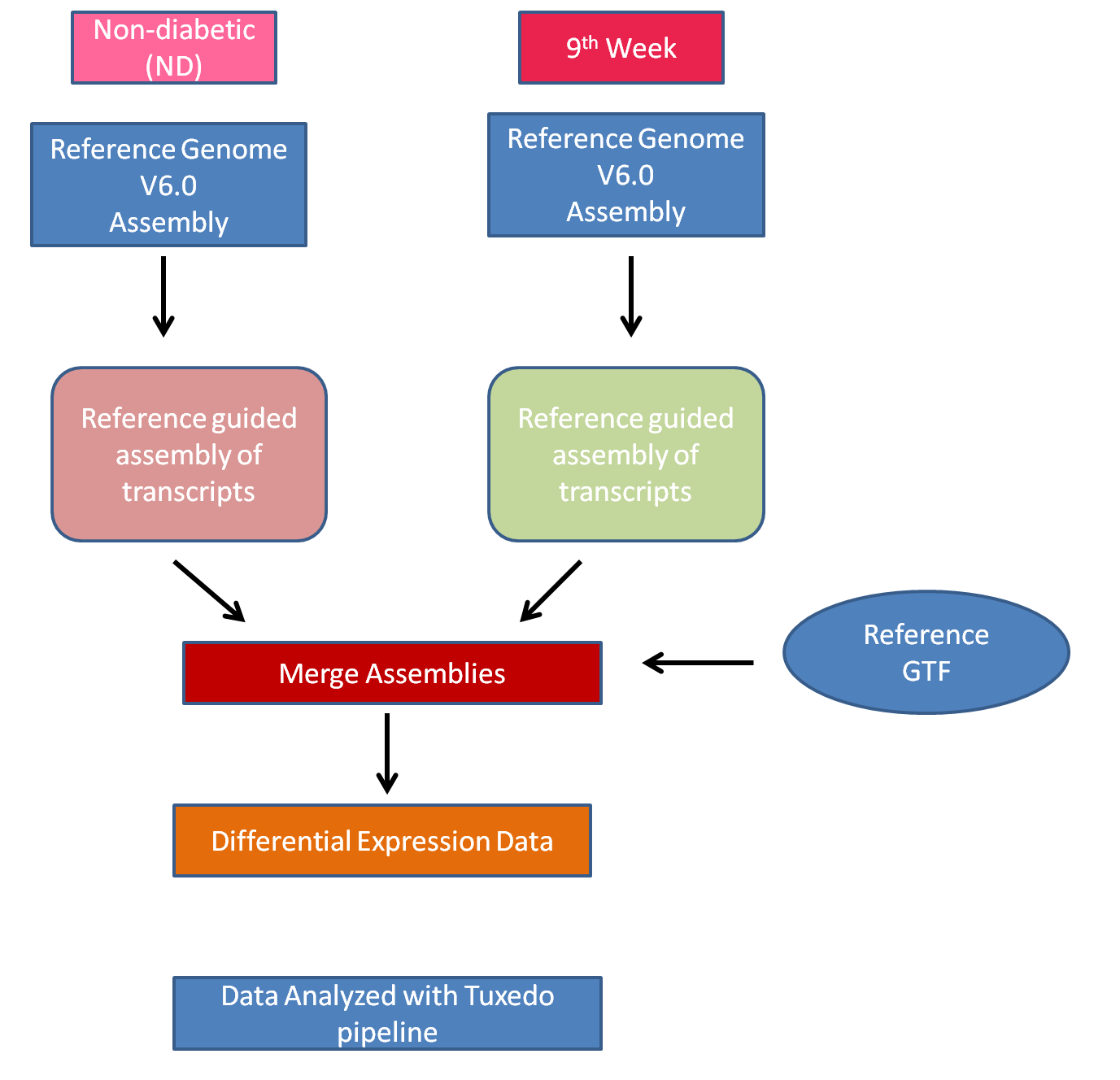
**


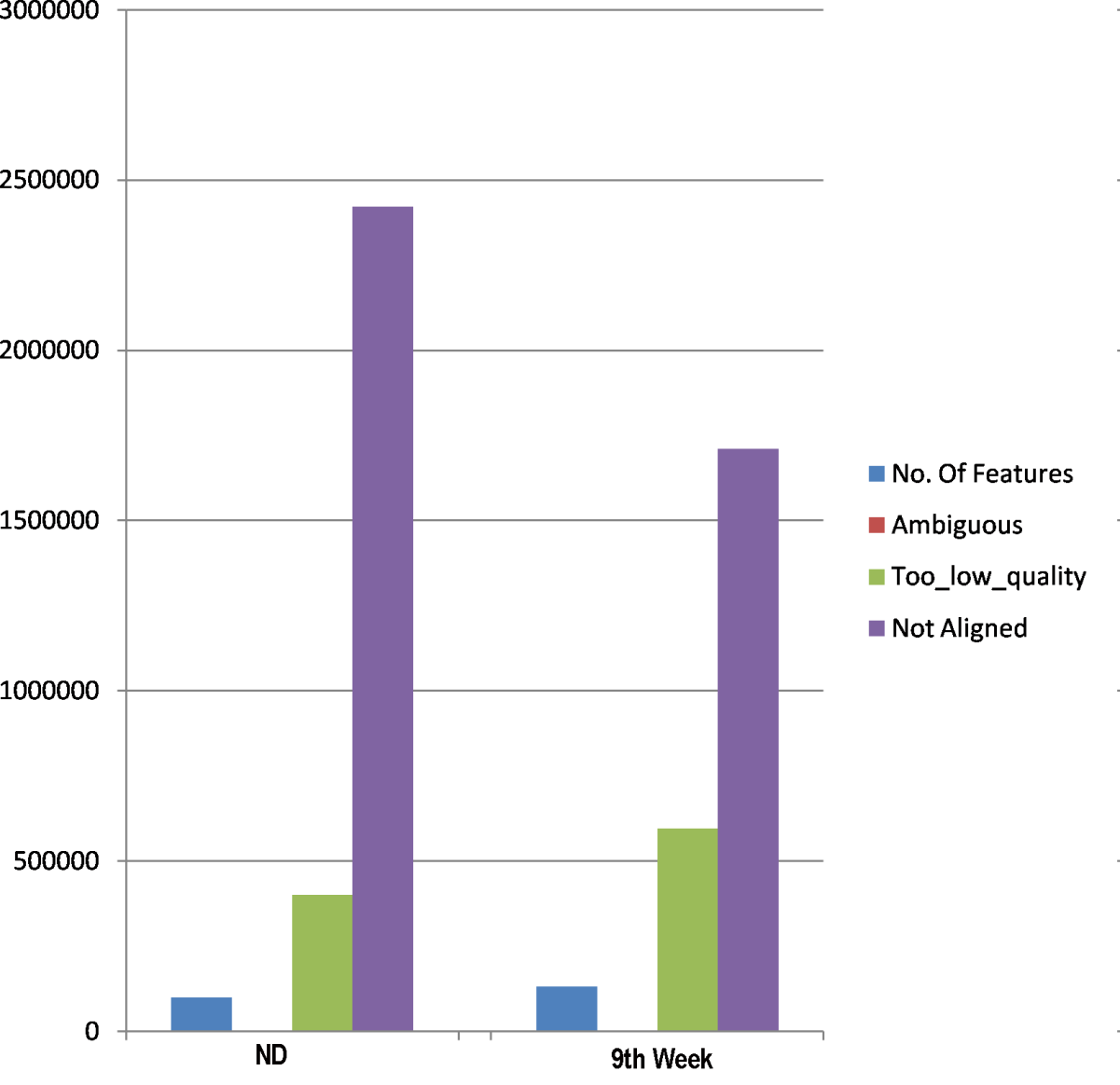
**Figure 3: Mapping statistics of samples against non-diabetic state (ND) and 9^th^ week of diabetes (9^th^)**

**Figure 4: Target region coverage of samples at non-diabetic state (ND) and 9^th^ week of diabetes (Ninth)**


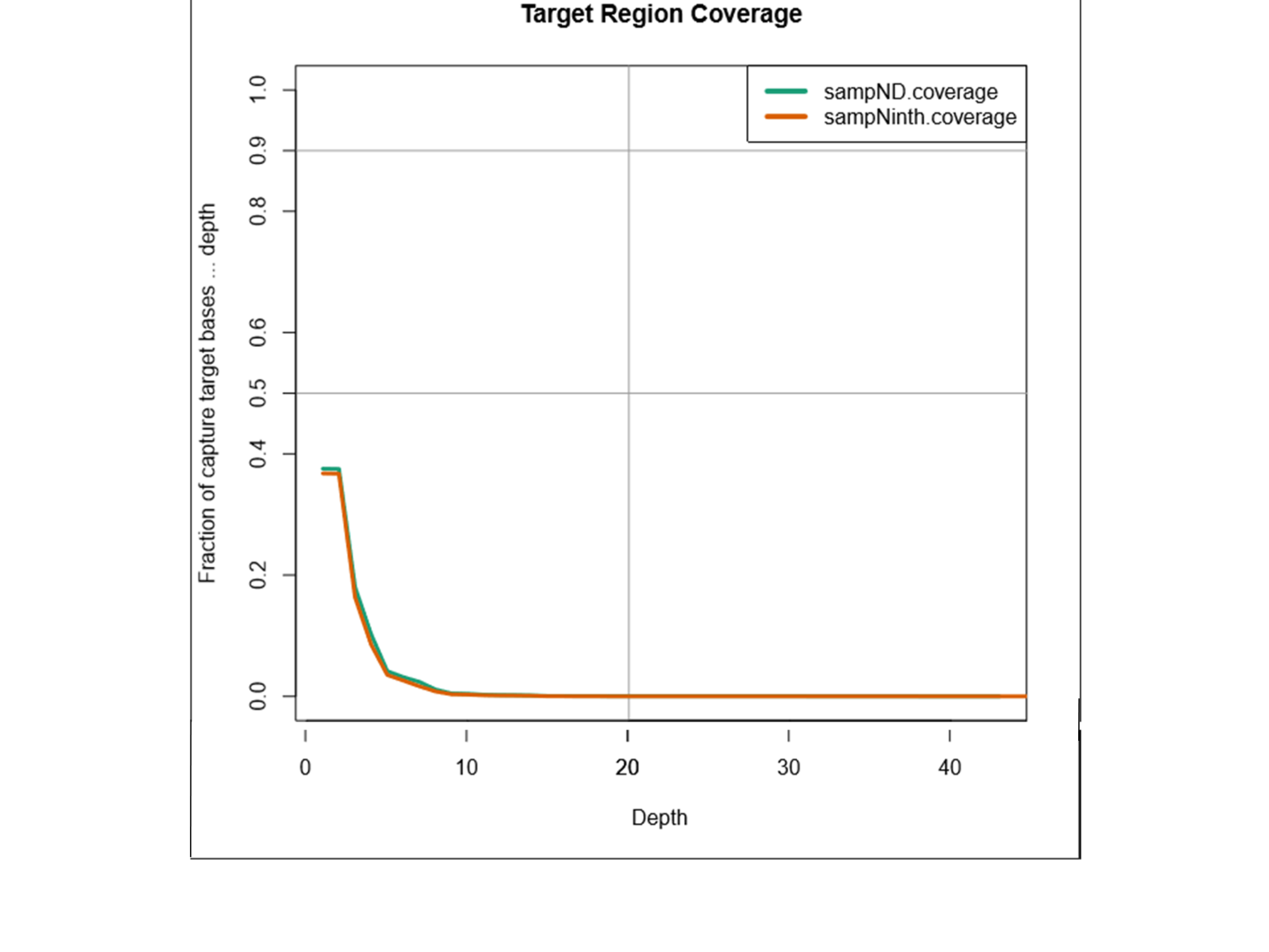

Supplement: S1 File — (DOC) [file pone.0154055.s001.doc]
