# Supplementary material for: Urinary Exosomal microRNA-451-5p Is a Potential Early Biomarker of Diabetic Nephropathy in Rats
Source: PLoS One. 2016 Apr 21;11(4):e0154055. doi: 10.1371/journal.pone.0154055 (PMC4839711; doi:10.1371/journal.pone.0154055)

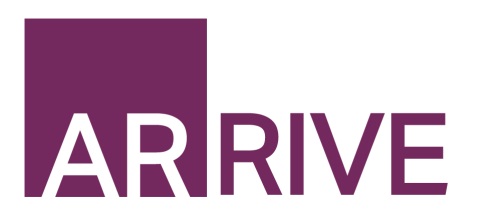


The ARRIVE Guidelines Checklist

Animal Research: Reporting In Vivo Experiments

Carol Kilkenny^1^, William J Browne^2^, Innes C Cuthill^3^, Michael Emerson^4^ and Douglas G Altman^5^

*^1^The National Centre for the Replacement, Refinement and Reduction of Animals in Research, London, UK, ^2^School of Veterinary Science, University of Bristol, Bristol, UK, ^3^School of Biological Sciences, University of Bristol, Bristol, UK, ^4^National Heart and Lung Institute, Imperial College London, UK, ^5^Centre for Statistics in Medicine, University of Oxford, Oxford, UK.*

|  | ITEM | RECOMMENDATION | Section/ Paragraph |
| --- | --- | --- | --- |
| 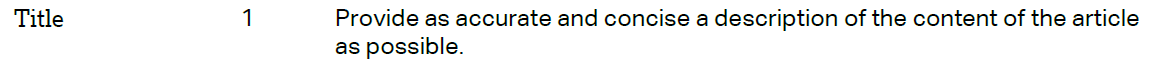 | | | Title |
| 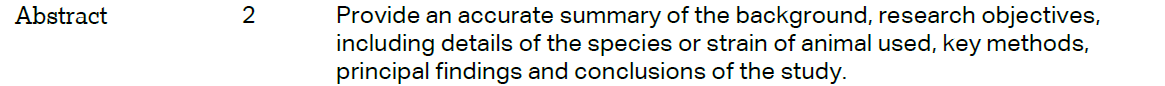 | | | Abstract |
| INTRODUCTION | | |  |
| 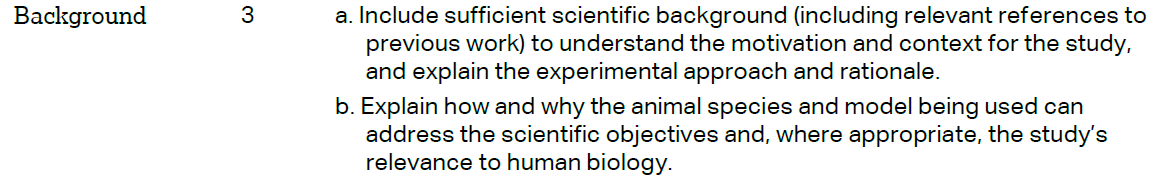 | | | Paragraph 1,2  Paragraph 2 |
| 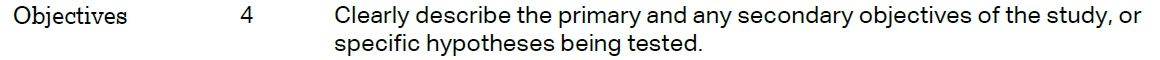 | | | Paragraph 2 |
| METHODS | | |  |
| 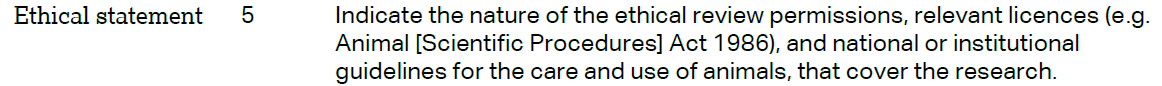 | | | Paragraph 1 |
| 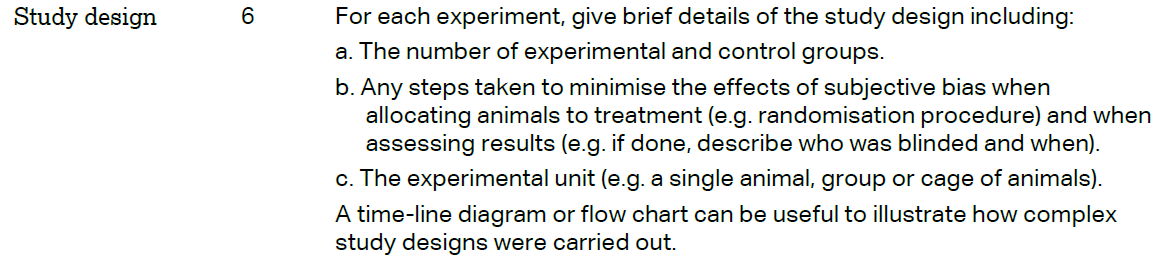 | | | Paragraph 1  Figure 1A |
| 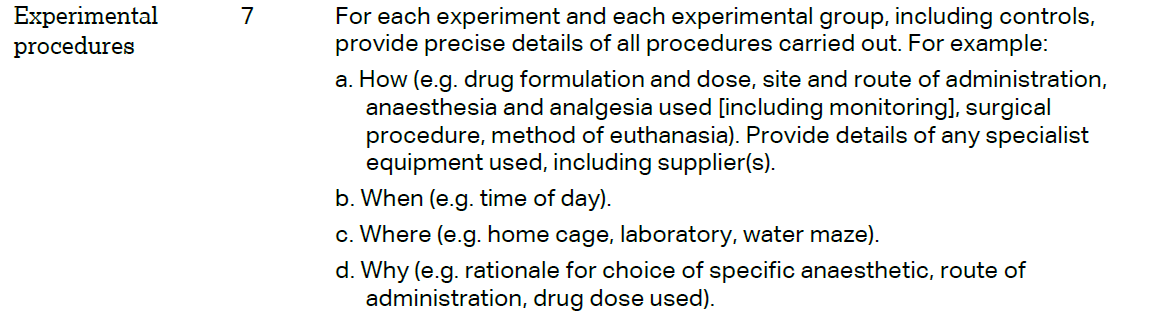 | | | Paragraph 1 |
| 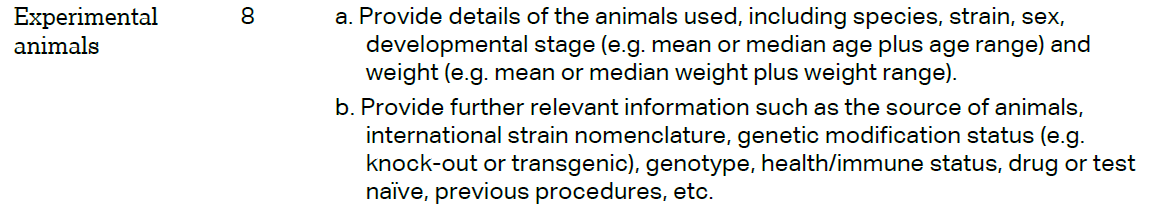 | | | Paragraph 1 |

The ARRIVE guidelines. Originally published in *PLoS Biology*, June 2010^1^

| 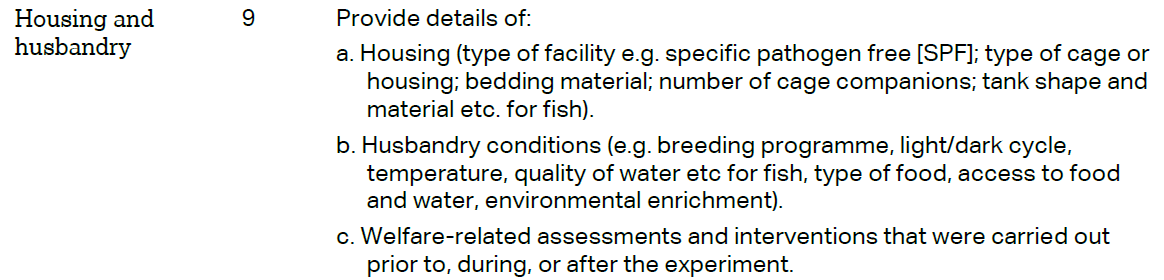 | Paragraph 1 | |
| --- | --- | --- |
| 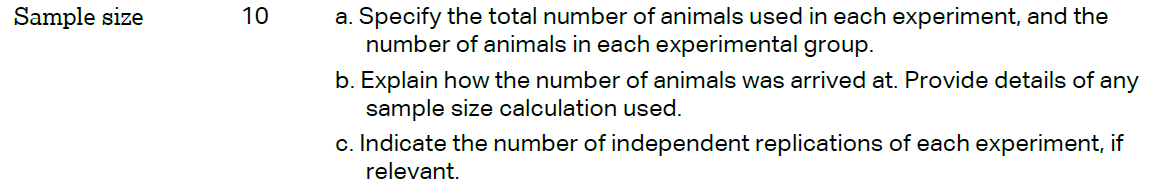 | Paragraph 1  None  Paragraph 9 | |
| 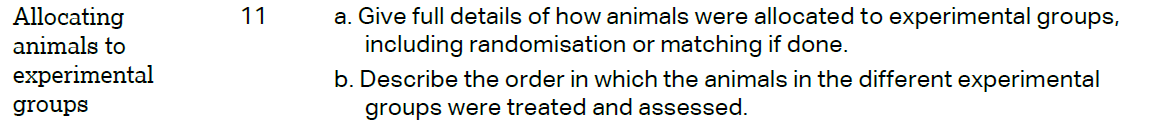 | Paragraph 1 | |
| 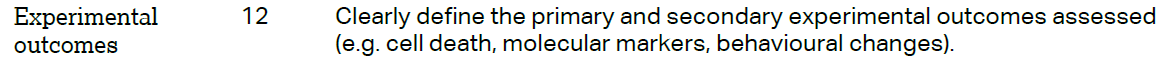 | Paragraph 2, 9, 11-13 | |
| 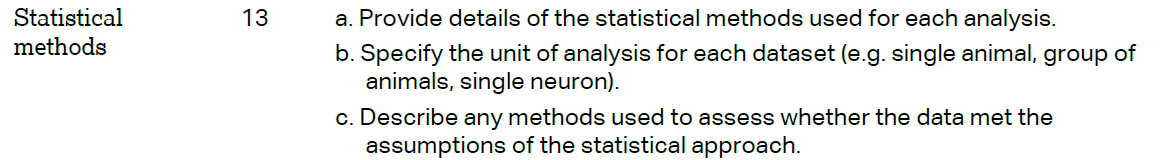 | Paragraph 14 | |
| RESULTS |  | |
| 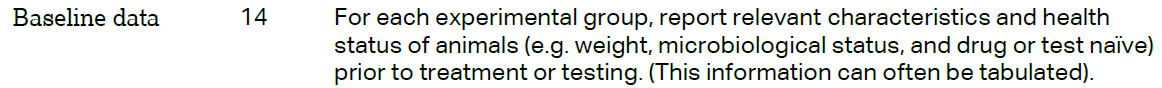 | Methods paragraph 1, Results paragraph 2,  Fig. 2 | |
| 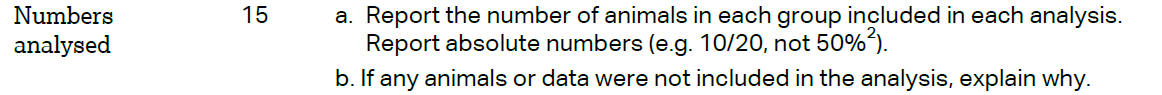 | Methods paragraphs 1, 6  ; Results paragraphs 3,4,7  Figure 2-7 | |
| 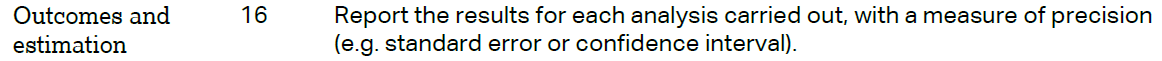 | Paragraphs 2,3,7  Figures 2-7 | |
| 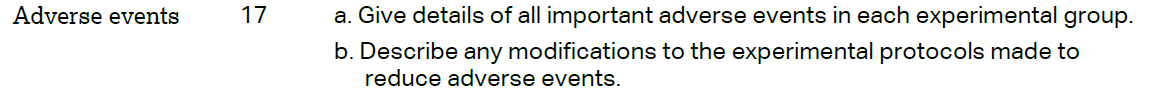 | None | |
| DISCUSSION |  | |
| 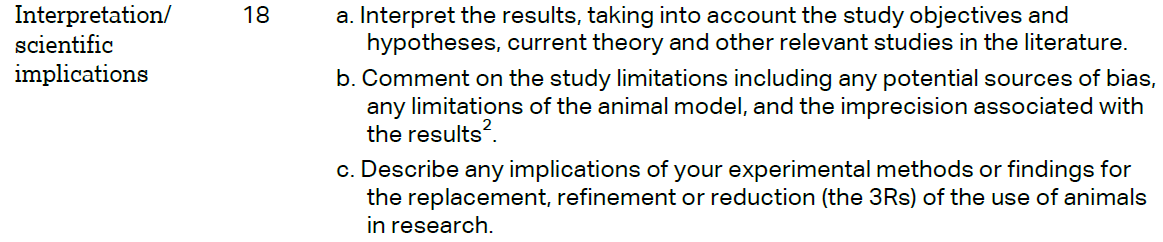 | Throughout  Paragraph 1,2,6  None | |
| 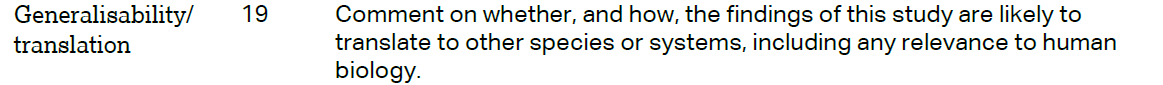 | Paragraph 2,6 | |
| 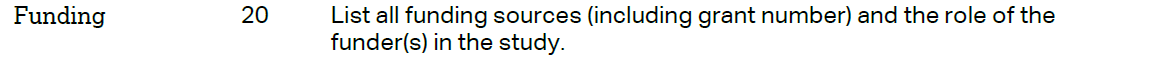 | Information submitted online |  |


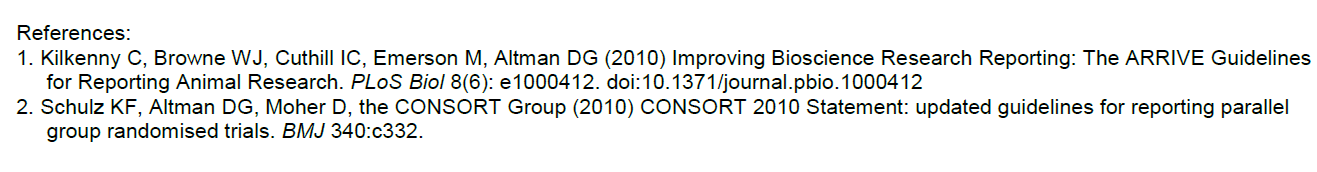

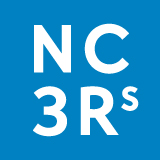

Supplement: S2 File — (DOC) [file pone.0154055.s002.doc]
