# Supplementary material for: Urinary Exosomal microRNA-451-5p Is a Potential Early Biomarker of Diabetic Nephropathy in Rats
Source: PLoS One. 2016 Apr 21;11(4):e0154055. doi: 10.1371/journal.pone.0154055 (PMC4839711; doi:10.1371/journal.pone.0154055)
Supplement: S3 Table — (DOC) [file pone.0154055.s005.doc]

**S3 Table**

|  | **CTRL** | | **DM** | | **DM + INS** | |
| --- | --- | --- | --- | --- | --- | --- |
|  | **Median** | **25^th^ -75^th^ percentile** | **Median** | **25^th^-75^th^ percentile** | **Median** | **25^th^ -75^th^ percentile** |
| **miR-451-5p** | | | | | | |
| 3^rd^ week | 0.0153 | 0.006-0.018 | 0.0002 | 0.000008- 0.050 | 0.00639 | 0.00152-0.0128 |
| 6^th^ week | 0.00543 | 0.003-0.03 | 0.183**^#^** | 0.067 - 35.416 | 0.0626 | 0.00116-0.0797 |
| 9^th^ week | 0.00716 | 0.00318-0.106 | 12.924**^#^** | 1.911 - 251496 | 0.115 | 0.0103- 0.904 |
| **miR-16** | | | | | | |
| 3^rd^ week | 8.8 | 3.856-11.244 | 40.024 | 7.815 - 204.5 | 13.072 | 1.124- 33.384 |
| 6^th^ week | 7.596 | 7.228-7.96 | 28.514 | 23.811- 582.1 | 6.764 | 3.504- 18.472 |
| 9^th^ week | 8.38 | 7.832-8.928 | 496.867**^#^** | 210.7- 540 | 14.728 | 1.972-27.48 |

**Change in Urinary exosomal miRNA-451-5p and miR-16 levels during the course of the study in rats** fold expression levels are represented as median values with 25^th^-75^th^ percentile in urinary exosomes from untreated diabetic (DM, n=10), non-diabetic control (CTRL, n=6) and insulin treated diabetic rats (DM + INS, n=6) at 3^rd^, 6^th^ and 9^th^ weeks post-injection . Fold expression was calculated using the 2^- ΔCT^ method, where ΔCT= CT_miRNA_ - CT_U6snRNA_. #p≤0.05 versus week 3 by paired t-test, (n=6-10/time point).
