# Supplementary material for: Expression of Concern: Urinary Exosomal microRNA-451-5p Is a Potential Early Biomarker of Diabetic Nephropathy in Rats
Source: PLoS One. 2024 Dec 23;19(12):e0316405. doi: 10.1371/journal.pone.0316405 (PMC11666015; doi:10.1371/journal.pone.0316405)
Supplement: S1 File — (PPTX) [file pone.0316405.s001.pptx]

## Slide 1
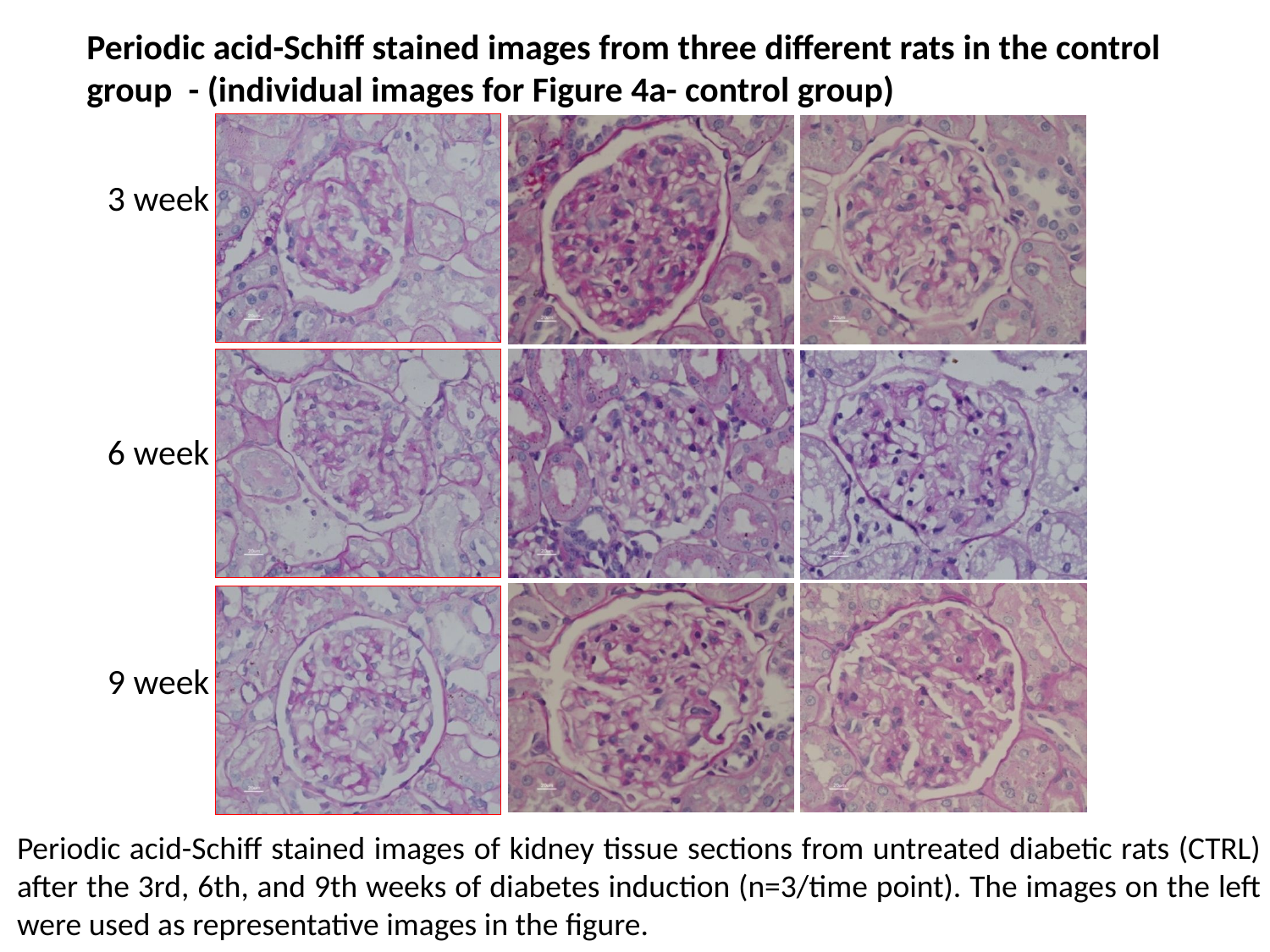

Periodic acid-Schiff stained images from three different rats in the control group - (individual images for Figure 4a- control group)
3 week
6 week
9 week
Periodic acid-Schiff stained images of kidney tissue sections from untreated diabetic rats (CTRL) after the 3rd, 6th, and 9th weeks of diabetes induction (n=3/time point). The images on the left were used as representative images in the figure.

## Slide 2
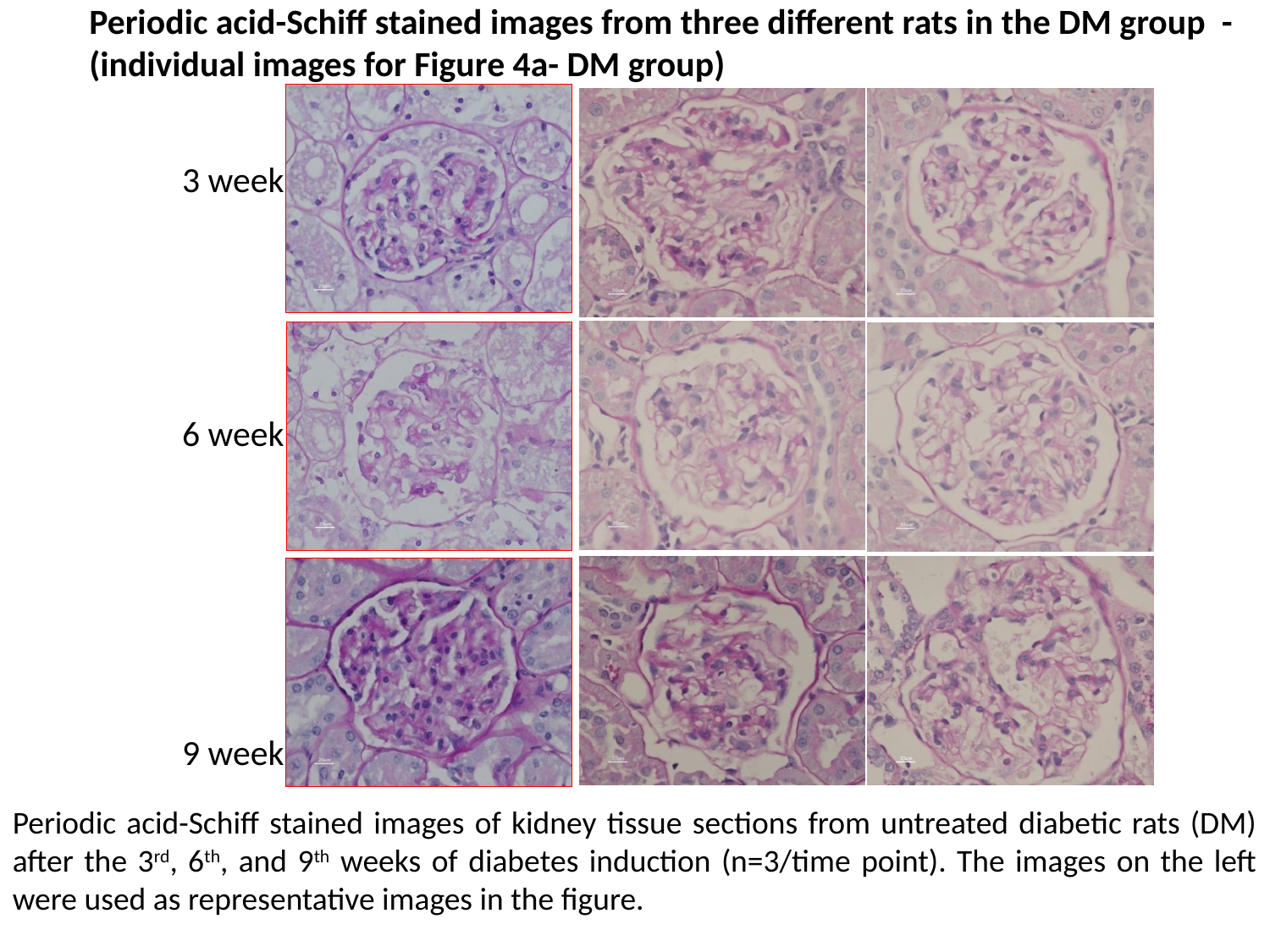

Periodic acid-Schiff stained images from three different rats in the DM group - (individual images for Figure 4a- DM group)
3 week
6 week
9 week
Periodic acid-Schiff stained images of kidney tissue sections from untreated diabetic rats (DM) after the 3rd, 6th, and 9th weeks of diabetes induction (n=3/time point). The images on the left were used as representative images in the figure.

## Slide 3
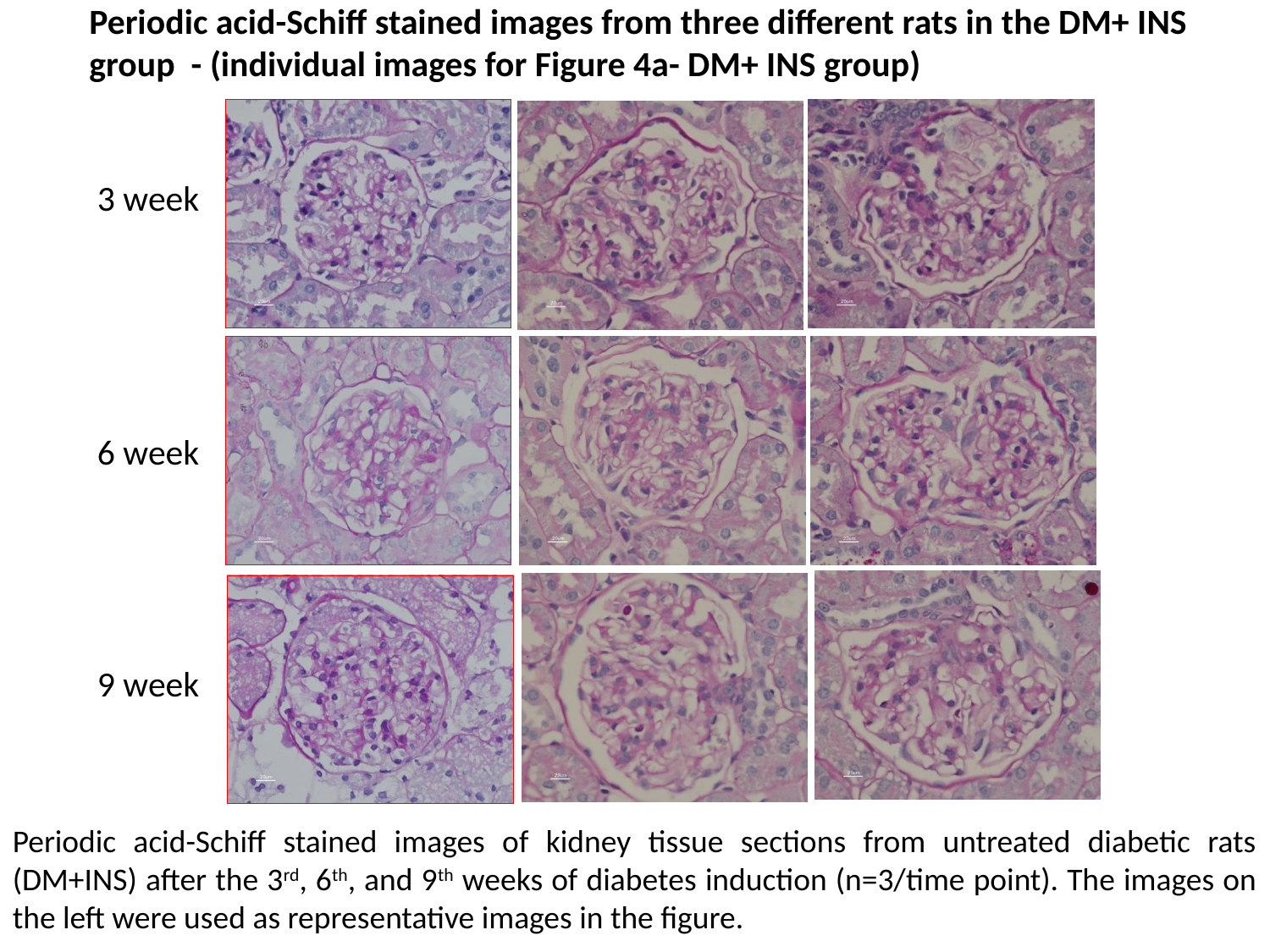

Periodic acid-Schiff stained images from three different rats in the DM+ INS group - (individual images for Figure 4a- DM+ INS group)
3 week
6 week
9 week
Periodic acid-Schiff stained images of kidney tissue sections from untreated diabetic rats (DM+INS) after the 3rd, 6th, and 9th weeks of diabetes induction (n=3/time point). The images on the left were used as representative images in the figure.

## Slide 4
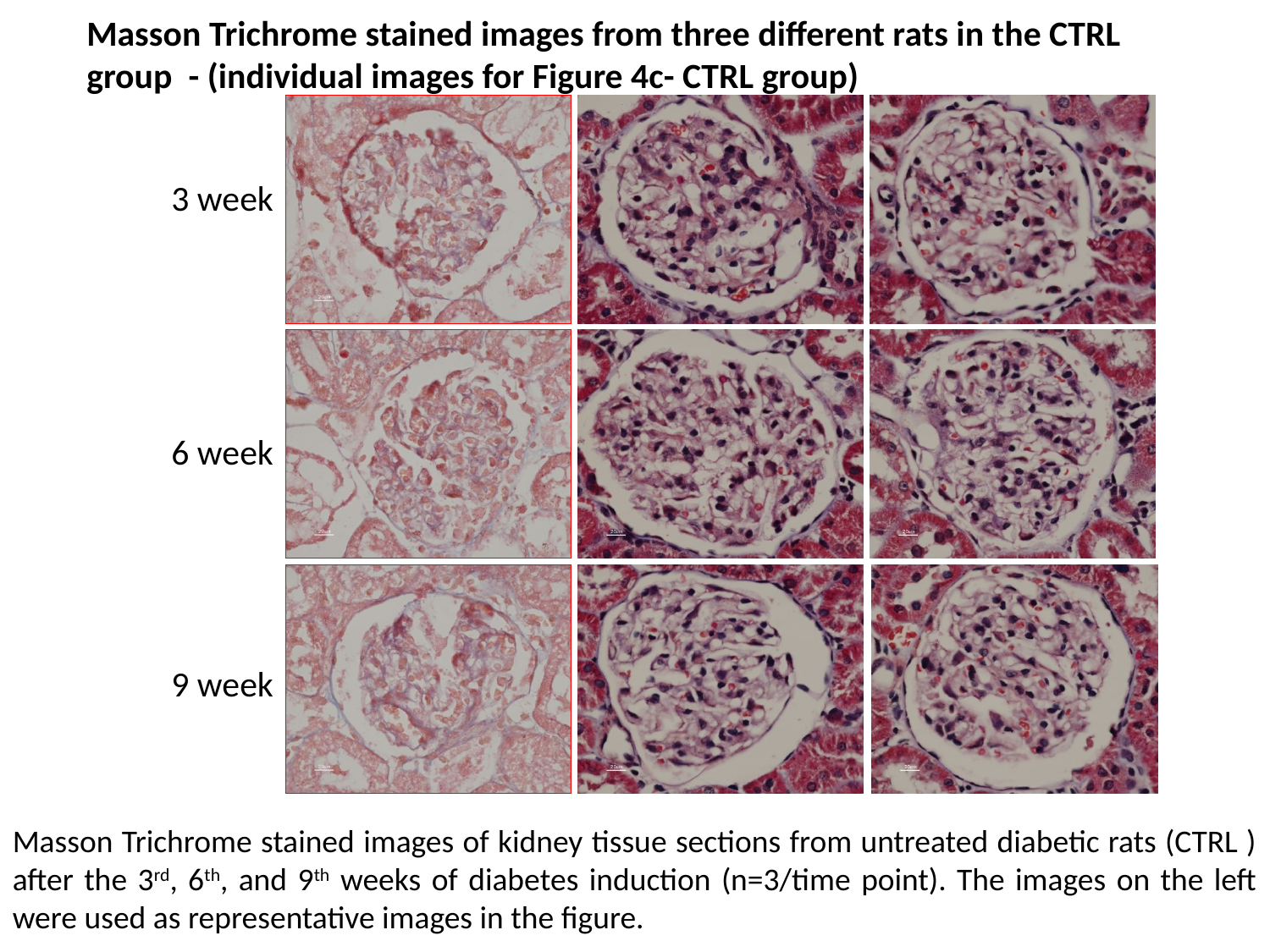

Masson Trichrome stained images from three different rats in the CTRL group - (individual images for Figure 4c- CTRL group)
3 week
6 week
9 week
Masson Trichrome stained images of kidney tissue sections from untreated diabetic rats (CTRL ) after the 3rd, 6th, and 9th weeks of diabetes induction (n=3/time point). The images on the left were used as representative images in the figure.

## Slide 5
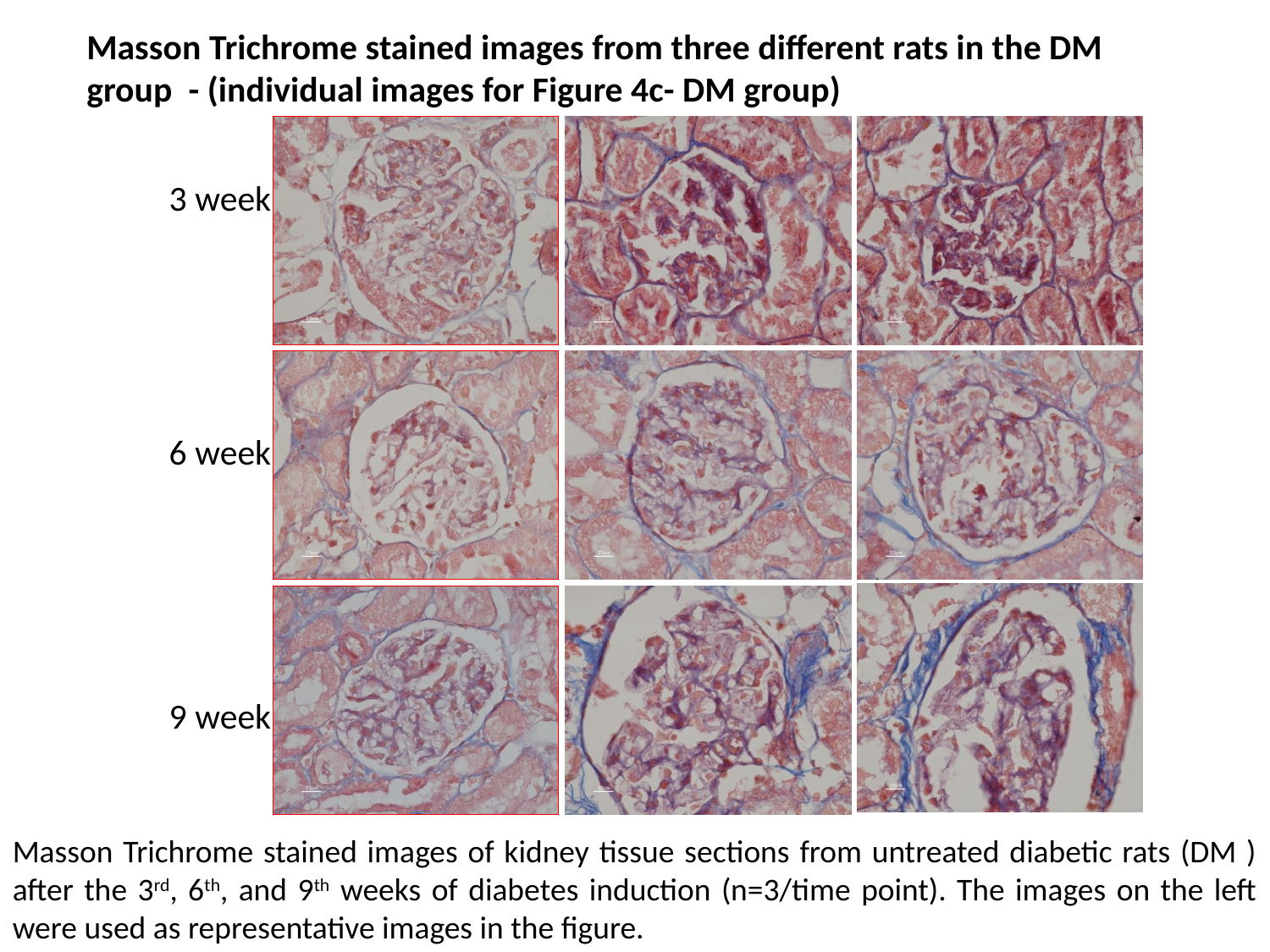

Masson Trichrome stained images from three different rats in the DM group - (individual images for Figure 4c- DM group)
3 week
6 week
9 week
Masson Trichrome stained images of kidney tissue sections from untreated diabetic rats (DM ) after the 3rd, 6th, and 9th weeks of diabetes induction (n=3/time point). The images on the left were used as representative images in the figure.

## Slide 6
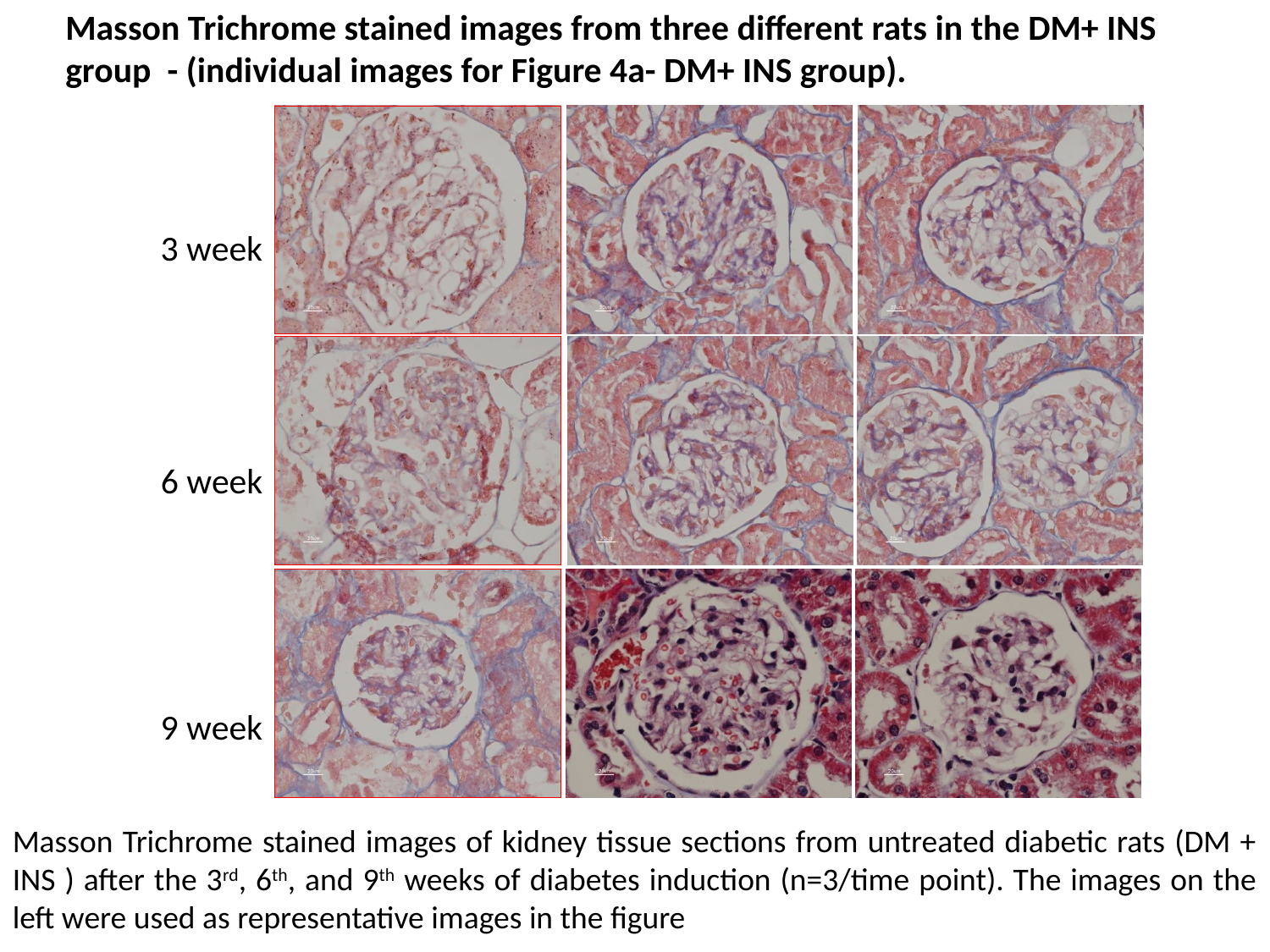

Masson Trichrome stained images from three different rats in the DM+ INS group - (individual images for Figure 4a- DM+ INS group).
3 week
6 week
9 week
Masson Trichrome stained images of kidney tissue sections from untreated diabetic rats (DM + INS ) after the 3rd, 6th, and 9th weeks of diabetes induction (n=3/time point). The images on the left were used as representative images in the figure
