# Supplementary material for: Expression of Concern: Urinary Exosomal microRNA-451-5p Is a Potential Early Biomarker of Diabetic Nephropathy in Rats
Source: PLoS One. 2024 Dec 23;19(12):e0316405. doi: 10.1371/journal.pone.0316405 (PMC11666015; doi:10.1371/journal.pone.0316405)
Supplement: S5 File — (DOC) [file pone.0316405.s005.doc]

**S3 Table**

|  | **CTRL** | | | **DM** | | **DM + INS** | | |
| --- | --- | --- | --- | --- | --- | --- | --- | --- |
|  | **Median** | **25^th^ -75^th^ percentile** | | **Median** | **25^th^-75^th^ percentile** | **Median** | **25^th^ -75^th^ percentile** | |
| **miR-451-5p** | | | | | | | | |
| 3^rd^ week | 0.01292 | 0.0063-0.01995 | | 0.0002 | 0.00001-0.01995 | 0.00395 | 0.00158-0.01258 | |
| 6^th^ week | 0.0045 | 0.00316-0.03162 | | 0.6046^#^ | 0.15135-5.12861 | 0.0395 | 0.00125-0.07943 | |
| 9^th^ week | 0.01027 | 0.00316-0.12589 | | 12.9245^#^ | 2.51188-1995.262 | 0.5134 | 0.01047-0.97723 | |
| **miR-16** | | | | | | | | |
| 3^rd^ week | 11.3869 | 10.00-15.8489 | 29.9033 | | 15.4881-95.4992 | 12.1627 | | 10.47129-24.54709 |
| 6^th^ week | 12.1627 | 12.0226-13.18257 | 25.81306 | | 10.47129-588.8436 | 9.55246 | | 8.1283-10.2329 |
| 9^th^ week | 11.6214 | 10.2329-12.30269 | 354.6877^#^ | | 154.8817-512.8614 | 18.43823 | | 17.378-19.49845 |

**Change in Urinary exosomal miRNA-451-5p and miR-16 levels during the course of the study in rats** fold expression levels are represented as median values with 25^th^-75^th^ percentile in urinary exosomes from untreated diabetic (DM), non-diabetic control (CTRL) and insulin treated diabetic (DM + INS) rats from study 1 at the 3^rd^, 6^th^ and 9^th^ weeks post-injection. Fold expression was calculated using the 2^- ΔCT^ method, where ΔCT= CT_miRNA_ - CT_U6snRNA_. #p≤0.05 versus week 3 by paired t-test on log transformed values, (n=6-10/time point).
